# Lack of Effect of Oral Sulforaphane Administration on Nrf2 Expression in COPD: A Randomized, Double-Blind, Placebo Controlled Trial

**DOI:** 10.1371/journal.pone.0163716

**Published:** 2016-11-10

**Authors:** Robert A. Wise, Janet T. Holbrook, Gerard Criner, Sanjay Sethi, Sobharani Rayapudi, Kuladeep R. Sudini, Elizabeth A. Sugar, Alyce Burke, Rajesh Thimmulappa, Anju Singh, Paul Talalay, Jed W. Fahey, Charles S. Berenson, Michael R. Jacobs, Shyam Biswal, on behalf of Broccoli Sprout Extract Trial Research Group

**Affiliations:** 1 Johns Hopkins University, School of Medicine, Baltimore, Maryland, United States of America; 2 Johns Hopkins Bloomberg School of Public Health, Baltimore, Maryland, United States of America; 3 Temple University, Philadelphia, Pennsylvania, United States of America; 4 University at Buffalo, SUNY and VA WNY Healthcare system, Buffalo, New York, United States of America; Central Michigan University College of Medicine, UNITED STATES

## Abstract

**Background:**

COPD patients have high pulmonary and systemic oxidative stress that correlates with severity of disease. Sulforaphane has been shown to induce expression of antioxidant genes via activation of a transcription factor, nuclear factor erythroid-2 related factor 2 (Nrf2).

**Methods:**

This parallel, placebo-controlled, phase 2, randomized trial was conducted at three US academic medical centers. Patients who met GOLD criteria for COPD and were able to tolerate bronchoscopies were randomly assigned (1:1:1) to receive placebo, 25 μmoles, or 150 μmoles sulforaphane daily by mouth for four weeks. The primary outcomes were changes in Nrf2 target gene expression (NQ01, HO1, AKR1C1 and AKR1C3) in alveolar macrophages and bronchial epithelial cells. Secondary outcomes included measures of oxidative stress and airway inflammation, and pulmonary function tests.

**Results:**

Between July 2011 and May 2013, 89 patients were enrolled and randomized. Sulforaphane was absorbed in the patients as evident from their plasma metabolite levels. Changes in Nrf2 target gene expression relative to baseline ranged from 0.79 to 1.45 and there was no consistent pattern among the three groups; the changes were not statistically significantly different from baseline. Changes in measures of inflammation and pulmonary function tests were not different among the groups. Sulforaphane was well tolerated at both dose levels.

**Conclusion:**

Sulforaphane administered for four weeks at doses of 25 μmoles and 150 μmoles to patients with COPD did not stimulate the expression of Nrf2 target genes or have an effect on levels of other anti-oxidants or markers of inflammation.

**Trial Registration:**

Clinicaltrials.gov: NCT01335971.

## Introduction

COPD, caused primarily by smoking, is the third leading cause of death in the US and worldwide [1,2]. Other than smoking cessation, there are few treatments that address the pathobiology of COPD. Evidence points to inflammation and increased oxidative stress in the lung as promoters of the clinical manifestations of COPD [3–6]. Therefore, one approach to therapy would be to stimulate the endogenous antioxidant defense mechanisms [7]. Nuclear factor erythroid-2-related factor 2 (Nrf2), a transcription factor activated by oxidative stress, acts to promote anti-oxidant enzymes that play key roles in cellular defenses [8]. Activation of Nrf2 protected mice from developing emphysema after chronic smoke exposure, decreased oxidative stress, increased proteasomal anti-apoptotic cytoprotective responses, and improved bacterial phagocytosis and killing [9–13]. Similarly, in human COPD lung cells, Nrf2 activation has been shown to decrease oxidative stress and improve bacterial clearance in macrophages [13,14]. Sulforaphane, a derivative of broccoli and other cruciferous vegetables, has been shown to stimulate Nrf2 activity *in vitro* and *in vivo* [15–20]. Thus, there is strong rationale for testing whether sulforaphane can target Nrf2 to decrease oxidative stress and inflammation in COPD patients. We have conducted a study to assess whether daily ingestion of sulforaphane by COPD patients for four weeks increased Nrf2 activity in alveolar macrophages and bronchial epithelial cells. Secondary outcomes included anti-oxidants concentrations and inflammatory markers in biospecimens, pulmonary function and patient-reported symptoms.

## Methods

### Study design and participants

This phase 2 trial was a multicenter, randomized, placebo-controlled, double masked, 3 arm parallel group trial designed to evaluate the effectiveness of oral sulforaphane on Nrf2 target gene expression and downstream anti-oxidants, and to determine safety and tolerability of two doses of sulforaphane. The study was conducted at 3 academic medical centers and the protocol (S1 Study Protocol) and consent statement were approved by the institutional review board (IRB) at each center; participant’s consent was documented in writing. The institutional review committees were: IRB-FC at Johns Hopkins Bloomberg School of Public Health for the data coordinating center and central laboratories, and IRB-2 at Johns Hopkins Medicine, Medical Interventions Committee A1 at Temple University, and the Buffalo VA Medical Center IRB for the 3 clinical centers, respectively; The trial was conducted under an Investigational New Drug Exemption (#109233) and was registered at Clinicaltrials.gov (NCT01335971). This report adheres to Consolidated Standards of Reporting Clinical Trials (CONSORT) guidelines for clinical trials (S1 CONSORT Checklist).

Active and former smokers aged 40 years or older with physician diagnosed COPD who were able to tolerate repeated bronchoscopies were enrolled. Eligible participants were required to have a smoking history of 10 or more pack-years, post bronchodilator FEV_1_/FVC ratio less than 0.70 and a percent predicted FEV_1_ of 40–80%. Participants agreed to ingest no more than one serving of cruciferous vegetables per week during the run-in and treatment periods. Patients were excluded from the study for any of the following: COPD exacerbation requiring treatment within the preceding six weeks; significant co-morbidities that would interfere with study participation or interpretation of the results; acute coronary syndrome or acute myocardial infarction within preceding six months; cancer other than skin or localized prostate within preceding five years; child-bearing potential with lack of adequate contraception; allergy to local anesthesia; resting hypoxemia; glomerular filtration rate less than 30 mL/min; liver enzymes four times upper limit of normal; or current use of warfarin.

### Randomization and masking

Participants were assigned to receive sulforaphane, extracted from broccoli sprouts, at 25 micromoles (4.4mg) or 150 micromoles (26.6 mg), or placebo (microcellulose) once daily by mouth. Treatment assignments were generated by computer and stratified by clinic with allocation ratio of 1:1:1 using a permuted block randomization scheme with variable blocks sizes prepared by the data coordinating center. Treatment assignments were concealed prior to randomization and were masked to the participants, clinic staff, central laboratory personnel and study data analysts. Clinical personnel keyed in eligibility data into a web-based treatment assignment program to enroll participants and received study drug kit identification numbers. Drug and placebo were supplied in similar appearing capsules, back-filled with methylcellulose to approximate similar appearance and weight. For quality assurance, sulforaphane levels in study drug kits stored at each site were measured four times during the study at approximately six months interval; the levels varied from -0.3% to -7.8% of expected level.

### Procedures

**Fig 1:**
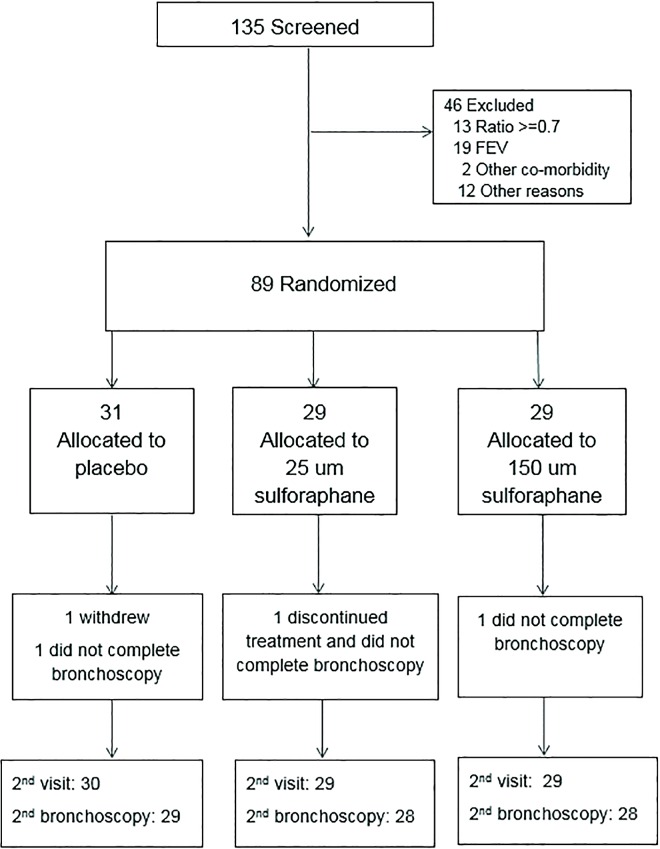
CONSORT Diagram.

Participants had five study visits over a six week period. Prior to randomization, participants were assessed for eligibility and baseline data were collected; bronchoscopy was performed on a separate day, usually on the day of randomization. At the final visit (target date four weeks after randomization) follow-up data and biospecimens were collected; the second bronchoscopy was performed on a separate day, usually the next day (Fig 1).

At study visits, participants provided data on medical history and COPD symptoms; completed the Saint George’s Respiratory Questionnaire (SGRC) and the American Thoracic Society-Division of Lung Disease Respiratory Questionnaire (ATA-DLD, baseline only); underwent a physical examination, pre and post bronchodilator spirometry, lung volume measurements, carbon monoxide diffusing capacity (DLCO) and pulse oximetry; and provided blood, urine and expired breath condensate specimens. Peripheral blood monocytes (PBMC) and plasma were isolated from blood. Expired breath condensate was collected via the R-tube method (Respiratory Research Inc, Austin, TX). Fiberoptic bronchoscopy was performed under sedation to collect endobronchial brushings and bronchoalveolar lavage to isolate alveolar macrophages and bronchial epithelial cells. Nasal brushings were obtained prior to bronchoscopy to isolate nasal epithelial cells. Participants were contacted by phone to reinforce instructions about study medication use and to check for adverse events following bronchoscopy. Sample processing procedures were standardized across all three centers by trained laboratory personnel certified on study procedures (S1 File). Hematology, serum chemistry and urinalysis were performed at local laboratories.

Specimens collected and processed at each center were shipped to the central laboratory for gene expression analysis. Total RNA was extracted from specimens using the RNeasy kit (Qiagen) and quantified by ultraviolet absorption spectrophotometry. Gene expression was evaluated using quantitative reverse transcription real-time polymerase chain reaction (qRT-PCR). The reverse transcription reaction was performed using a high capacity cDNA synthesis kit (Sensiscript RT kit (Qiagen)). Quantitative real time RT-PCR analyses of Nrf2, its inhibitor, Kelch like ECH associated protein-1 (KEAP1) and several target genes (NQ01, HO1, AKR1C1, AKR1C3, and secretory leukoprotease inhibitor (SLPI)) were performed by using assay-on-demand primers and probe sets from Applied Biosystems.

### Outcomes

The primary outcomes were fold-change from baseline in Nrf2 target gene expression (NQ01, HO1, AKR1C1, AKR1C3) at four weeks in alveolar macrophages and bronchial epithelial cells. In addition, we evaluated expression of other genes in the Nrf2/Keap1 pathway (Nrf2, KEAP1, and SLPI), and markers of anti-oxidant activity and inflammation in alveolar macrophages, bronchial epithelial cells, nasal epithelial cells, serum and PBMCs. Markers of oxidative stress included isoprostane, thiobarbituric acid reactive substances (TBARS) in plasma and expired breath condensate; and cytokine profiles in bronchoalveolar lavage (BAL) fluid. Other measures included spirometry and patient reported outcomes (Medical Research Council (MRC) Dyspnea scale and SGRQ). Key safety outcomes included treatment emergent and serious adverse events, serum chemistry measures, complete blood counts, and thyroid stimulating hormone.

### Statistical analysis

The trial was designed to enroll 90 participants (30 per group) in order to achieve 80% power to detect a 0.41 fold-change (141% of baseline) in Nrf2 target gene expression in alveolar macrophages and bronchial epithelial cells with a two-sided type 1 error rate of 0.01 to account for multiple comparisons, i.e., placebo versus 25 micromoles and placebo versus 150 micromoles in alveolar macrophages and bronchial epithelial cells. The standard deviation estimate of 1.01 was based on estimates from assays of human COPD lung tissues and nasal epithelial cells [21].

**Fig 2:**
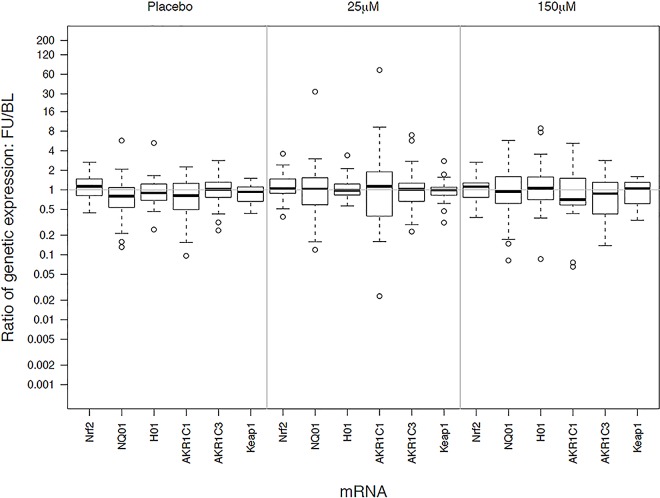
Relative changes in gene expression by treatment group in alveolar macrophages Treatments are labeled as placebo (no sulforaphane), 25 μM) and 150 μM of sulforaphane. Gene identifiers are listed along the x-axis. The y-axis displays the distribution of fold-change in gene expression (follow-up divided by baseline).

**Fig 3:**
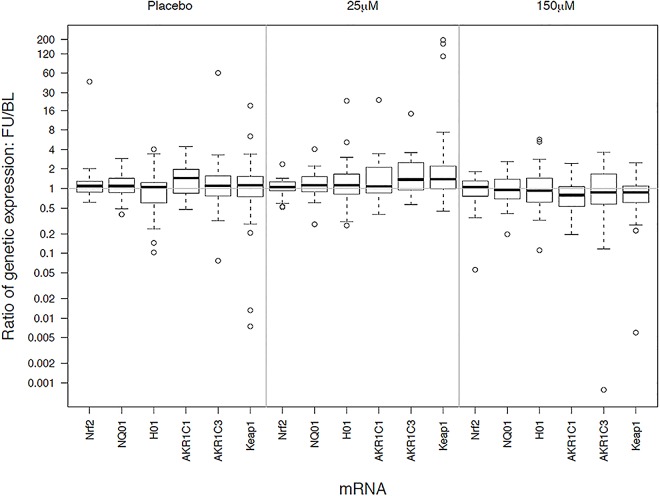
Relative changes in gene expression by treatment group in bronchial epithelial cells. Treatments are labeled as placebo (no sulforaphane), 25 μM) and 150 μM of sulforaphane. Gene identifiers are listed along the x-axis. The y-axis displays the distribution of fold-change in gene expression (follow-up divided by baseline).

**Fig 4:**
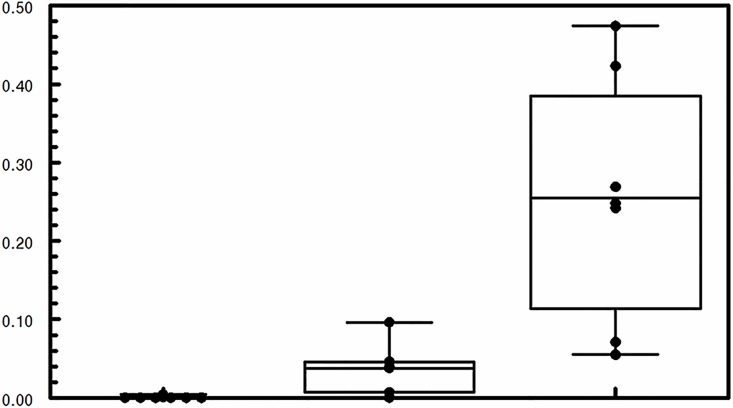
Dithiocarbamate (DTC) concentration in 21 randomly selected plasma samples. Treatments are labeled as placebo (no sulforaphane), 25 μM, and 150 μM of sulforaphane.

The comparability of the participant characteristics were examined (Tables 1 and 2). The primary analysis was an intention-to-treat analysis of fold-change in Nrf2 target gene expression with sulforaphane dose as the main effect, Kruskal-Wallis tests were used to evaluate treatment differences (Tables 3 and 4 and S1–S3 Tables). Relative gene expression (Table 3 and Figs 2 and 3) was quantified using the comparative CT method [22]. The expression of a target gene was quantified relative to the expression of a reference gene (β-actin was the endogenous control used for all specimen types) and a "fold change" in expression was calculated comparing the relative expression of the target at follow-up compared to baseline. Similar methods were used to evaluate the effect of sulforaphane dose on other phase II antioxidant gene expression (Table 3) and inflammatory markers (Table 4). P-values were not adjusted for multiple comparisons. Chi-square were used to test for differences among the treatment groups in patient-reported side symptoms (S4 Table). Dithocarbamate (DTC) levels were measured in plasma using an established methodology (Fig 4) [23]. Treatment effects among subgroups of interest (smoking status and GOLD stage) were examined. All analyses were conducted according to treatment assignment using all available data. Data were analyzed using SAS (version 9.3). The trial was monitored by an independent data monitoring board that met five times over the course of the study; no interim analyses of outcome data by treatment group were planned or conducted during the study.

**Table 1:** Baseline characteristics of randomized participants by treatment group.

	*Sulforaphane Dose Group*			
	*Placebo*	*25* μ*moles*	*150* μ*moles*	*Total*
N randomized	31	29	29	89
Years of age, median (IQR)	59 (52–67)	59 (54–65)	56 (52–62)	58(54–65)
Male, n (%)	16 (52%)	17 (59%)	21 (72%)	54 (61%)
**Race or ethnic group,** n (%)				
White	20 (65%)	16 (55%)	15 (52%)	51 (57%)
Black	11 (35%)	13 (45%)	14 (48%)	38 (43%)
Hispanic	0 (0%)	1 (3%)	0 (0%)	1 (1%)
**COPD Characteristics n (%)**				
10 or more pack years of smoking history	30 (100%)	29 (100%)	29 (100%)	88 (100%)
Smoke cigarettes now	20 (65%)	16 (55%)	18 (62%)	54 (61%)
Smoke 10 or more cigarettes a day now	10 (32%)	9 (31%)	10 (34%)	29 (33%)
COPD exacerbation in prior 12 months	5 (16%)	7 (24%)	7 (24%)	19 (21%)
**Pulmonary Function Measures (post-BD) median (IQR)**				
Post bronchodilator FEV1 (%predicted)	61 (54–70)	54 (50–65)	65 (55–72)	61 (53–70)
Post bronchodilator FEV1/FVC ratio	0.56 (0.51–0.63)	0.52 (0.47–0.59)	0.57 (0.50–0.63)	0.56 (0.48–0.62)
DLCO (mL/mmHg/min)	14.8 (11.8–19.5)	15.7 (12.1–19.3)	16.3 (12.3–21.4)	15.7 (12.1–20.9)
TLC (Liters)	6.0 (4.7–7.3)	5.5 (4.9–6.8)	6.3 (5.7–7.0)	6.0 (5.0–7.2)
SVC (Liters)	3.1 (2.4–4.1)	3.0 (2.8–3.7)	3.7 (3.1–4.4)	3.3 (2.7–4.1)
FRC (Liters)	3.6 (3.1–4.5)	3.6 (3.0–4.1)	3.4 (3.0–3.9)	3.5 (3.0–4.2)
RV (Liters)	2.6 (2.5–3.4)	2.6 (2.2–3.5)	2.7 (2.3–2.9)	2.6 (2.2–3.2)
Pulse oximetry (%)	95 (94–97)	96 (95–97)	96 (94–97)	96 (94–97)
**Use of respiratory medications in prior 2 weeks, n (%)**				
Short acting beta-agonist (SABA)	22 (71%)	21 (72%)	18 (62%)	61 (69%)
Short-acting anticholinergic bronchodilator	1 (3%)	1 (3%)	2 (7%)	4 (4%)
SABA and short-acting anticholinergics	3 (10%)	1 (3%)	4 (14%)	8 (9%)
Long-acting beta-agonist (LABA)	2 (6%)	1 (3%)	2 (7%)	5 (6%)
Inhaled corticosteroids	2 (6%)	2 (7%)	3 (10%)	7 (8%)
LABA and inhaled corticosteroid	14 (45%)	13 (45%)	13 (45%)	40 (45%)
Long-acting anticholinergic bronchodilator	7 (23%)	9 (31%)	10 (34%)	26 (29%)
Leukotriene modifiers	0 (0%)	2 (7%)	2 (7%)	4 (4%)
Aspirin	7 (23%)	7 (24%)	8 (28%)	22 (25%)
Anticoagulants or Others (Warfarin, Clopidogrel, Dabigatran, other)	2 (6%)	0 (0%)	1 (3%)	3 (3%)
**Medical Research Council Dyspnea Score median (IQR)**	2(1–3)	2(2–3)	2(1–3)	2(1–3)
**St Georges Respiratory questionnaire median (IQR)**				
Total score	43 (24–58)	47 (27–56)	39 (24–54)	40 (26–56)
Symptoms score	58 (32–70)	55 (43–68)	50 (31–57)	52 (36–68)
Activity score	54 (30–74)	60 (47–74)	50 (41–73)	55 (36–74)
Impacts score	27 (17–44)	34 (15–46)	26 (13–42)	28 (15–44)
**Other self-reported co-morbidities, n (%)**				
Cardiac	3 (10%)	2 (7%)	4 (14%)	9 (10%)
Stroke	0 (0%)	2 (7%)	1 (3%)	3 (3%)
Obstructive sleep apnea	4 (13%)	2 (7%)	8 (28%)	14 (16%)
Diabetes	7 (23%)	2 (7%)	6 (21%)	15 (17%)
High blood pressure	19 (61%)	16 (55%)	17 (59%)	52 (58%)
Hepatitis/liver disease	3 (10%)	2 (7%)	4 (14%)	9 (10%)
Neurological	1 (3%)	3 (10%)	0 (0%)	4 (4%)
Psychological	8 (26%)	6 (21%)	5 (17%)	19 (21%)
Cancer	3 (10%)	2 (7%)	3 (10%)	8 (9%)
Other condition*	19 (61%)	16 (55%)	18 (62%)	53 (60%)

Abbreviations: IQR = interquartile range

**Gout, kidney disease, rheumatoid arthritis and other

**Table 2:** Baseline measures of antioxidants and markers of inflammation by treatment group.

Marker	*Sulforaphane Dose Group*		
	*Placebo*	*25* μ*moles*	*150* μ*moles*
	*Median (Interquartile Range)*		
**Serum** (N)	30	28	29
C-reactive protein (mg/L)	9.0 (2.6–11.6)	6.1 (3.0–12.5)	6.3 (2.8–10.9)
Interleukin-6 (pg/mL)	2.3 (1.8–3.6)	2.7 (1.4–3.7)	1.6 (1.2–2.6)
Interleukin-8 (pg/mL)	12.0 (9.8–15.6)	11.7 (8.1–14.2)	11.0 (8.7–18.6)
**Bronchial Alveolar Lavage** (N)	27	28	28
Interleukin-8 (pg/mg)	1.7 (0.8–5.4)	2.5 (1.3–4.3)	2.1 (0.5–3.6)
SLPI (pg/mg)	305 (179–455)	381 (226–456)	322 (219–480)
**Expired Breath Condensate** (N)	30	27	28
Isoprostane (ng/mg)	13.3 (6.2–28.0)	18.8 (7.9–55.2)	19.3 (6.6–34.6)
**Plasma** (N)	30	28	29
Isoprostane (ng/mg)	169 (111–336)	164 (73–400)	200 (90–526)
TBARS (nmol MDA/mL)	7.2 (5.6–9.3)	7.8 (5.8–8.8)	7.7 (6.3–8.8)
Total antioxidants (mM Trolox equivalents/L)	0.64 (0.59–0.68)	0.61 (0.57–0.67)	0.62 (0.55–0.66)

Abbreviations: TBARS = thiobarbituric acid reactive substances; SLPI = secretory leukoprotease inhibitor

**Table 3:** Fold-change* in genetic expression in bronchial epithelial cells and alveolar macrophages by treatment group.

		*Sulforaphane Dose Group*					
Gene	N	*Placebo*	*25* μ*moles*		*150* μ*moles*		*P-value†*
		*Median (Interquartile Range)*					
**Alveolar macrophages**							
NQ01	81	0.80 (0.53–1.09)		1.03 (0.56–1.60)		0.94 (0.59–1.72)	0.45
HO1	81	0.90 (0.69–1.34)		0.98 (0.83–1.31)		1.06 (0.68–1.74)	0.40
AKR1C1	81	0.81 (0.46–1.27)		1.13 (0.38–1.99)		0.71 (0.56–1.57)	0.75
AKR1C3	81	1.03 (0.76–1.37)		1.02 (0.67–1.31)		0.87 (0.40–1.32)	0.49
Nrf2	81	1.14 (0.79–1.52)		1.05 (0.87–1.47)		1.13 (0.74–1.28)	0.88
Keap1	81	0.94 (0.66–1.17)		0.99 (0.82–1.11)		1.06 (0.59–1.32)	0.71
**Bronchial epithelial cells**							
NQ01	82	1.09 (0.83–1.50)		1.12 (0.89–1.53)		0.96 (0.65–1.41)	0.69
HO1	84	1.05 (0.60–1.23)		1.12 (0.82–1.67)		0.93 (0.62–1.45)	0.53
AKR1C1	81	1.45 (0.84–1.98)		1.08 (0.85–2.14)		0.79 (0.53–1.08)	< .01
AKR1C3	81	1.10 (0.74–1.62)		1.38 (0.91–2.64)		0.87 (0.50–1.68)	0.06
Nrf2	83	1.09 (0.88–1.30)		1.06 (0.92–1.28)		1.06 (0.76–1.31)	0.68
** PBMC**							
NQ01	85	0.88 (0.74–1.45)		1.17 (0.82–1.82)		1.29 (0.72–2.01)	0.31
HO1	86	1.09 (0.94.1.08)		0.92 (0.73–1.42)		1.10 (0.78–1.50)	0.10
AKR1C1	86	1.10 (0.49–1.98)		1.00 (0.49–1.76)		0.94 (0.71–2.39)	0.79
AKR1C3	87	1.03 (0071–1.30)		0.90 (0.68–1.45)		1.14 (0.80–2.07)	0.21
Nrf2	85	0.94 (0.74–1.08)		1.17 (0.82–1.82)		0.96 (0.76–1.28)	0.21

*Follow-up expression relative to baseline expression

†P-value based on Kruskal-Wallis test

**Table 4:** Fold-change* in inflammatory marker concentrations by treatment group.

Marker	*Sulforaphane Dose Group*			
	*Placebo*	*25* μ*moles*	*150* μ*moles*	*P-value†*
	*Median (Interquartile Range)*			
**Serum (N)**	30	28	29	
C-reactive protein (mg/L)	0.99 (0.86–1.22)	0.90 (0.69–1.06)	1.01 (0.72–1.22)	0.41
Interleukin-6 (pg/mL)	0.75 (0.65–1.19)	0.90 (0.76–1.08)	1.12 (0.88–1.37)	0.07
Interleukin-8 (pg/mL)	1.06 (0.86–1.32)	1.04 (0.87–1.17)	1.03 (0.83- .21)	0.65
**Bronchial Alveolar Lavage (N)**	27	28	28	
Interleukin-8 (pg/mg)	1.22 (0.68–2.75)	0.94 (0.52–2.22)	1.11 (0.42–2.54)	0.71
SLPI (pg/mg)	1.51 (0.83–1.90)	1.09 (0.85–1.49)	1.12 (0.65–1.51)	0.33
**Expired Breath Condensate (N)**	30	27	28	
Isoprostane (ng/mg)	1.18 (0.42–1.79)	0.83 (0.24–1.41)	0.64 (0.29–1.33)	0.20
**Plasma (N)**	30	28	29	
Isoprostane (ng/mg)	0.89 (0.55–1.22)	0.90 (0.63–1.74)	0.88 (0.55–1.37)	0.80
TBARS (nmol MDA/ml)	0.96 (0.77–1.19)	1.05 (0.88–1.17)	1.06 (0.84–1.27)	0.35
Total antioxidants (mM Trolox equivalents/L)	0.97 (0.92–1.03)	0.92 (0.85–1.03)	0.97 (0.90–1.04)	0.53

Abbreviations: TBARS = thiobarbituric acid reactive substances; SLPI = secretory leukoprotease inhibitor

*Follow-up expression relative to baseline expression

†P-value based on Kruskal-Wallis test

### Role of the funding source

This study was funded by NIH/NHLBI (Grant Number U01HL105569). The sponsor had no role in study design, data collection, data analysis, data interpretation or writing of the report.

## Results

Between July 2011 and 29 May 2013,135 individuals were assessed for eligibility (Fig 1); 46 were excluded either at screening or during the run-in period. Eighty-nine participants who met the eligibility criteria were randomized into one of the three treatment groups. Only one participant, assigned to placebo, did not complete the study. Follow-up was completed on 13 June 2013. Three participants, one from each treatment group, did not complete the second bronchoscopy at four weeks. Compliance to study medications, based on capsule counts from returned blister-packs, ranged from 98 to 99%.

The baseline characteristics of the participants are shown in Table 1. Baseline demographics and COPD characteristics were similar among the three treatment groups. Most participants were white (57%) and male (61%); the median age at randomization was 58 years. Sixty-one percent of participants were current smokers at baseline, 21% had at least one unscheduled health care visit for COPD in the year previous to enrollment. The median post bronchodilator FEV_1_/FVC ratio was 0.56 and the median percent predicted post bronchodilator FEV_1_ was 61%. The majority (58%) reported having hypertension. None of the participants were receiving supplemental oxygen but many had used a short acting beta-agonist (69%) in the previous two weeks and most were being treated with inhaled corticosteroids, long-acting beta-agonists or both (82%). Measures of inflammation and antioxidants at baseline were also similar across the three treatment groups (Table 2). There were no differences among the treatment groups on Nrf2 gene expression relative to β-actin expression at baseline (data not shown).

The Nrf2 target gene expression in alveolar macrophages and bronchial epithelial cells did not significantly increase from baseline in any of the treatment groups, all of the 95% confidence intervals for the ratios of post to pre-treatment gene expression included 1.0 (Table 3, Figs 2 and 3). All but one of the treatment comparisons were similarly null, i.e., there were no differences among the treatment groups. The one nominally statistically significant difference among the treatment groups was for AKR1C1 expression in bronchial epithelial cells (P<0.01). AKR1C1 expression had the largest fold-change in the placebo group, 1.45, versus fold-changes of 1.08 and 0.79 in the 25μmoles and 150 μmoles groups, respectively; however none of these were significantly different from baseline value of the groups. Expression of the other Nrf2 target genes did not exhibit statistically significant increases from baseline or differences among treatment groups (Table 3, Figs 2 and 3). Subgroup analysis showed no treatment differences by GOLD stage, smoking status (current or former) or in post-hoc subgroups defined by increase or decrease bronchodilator response or FEV_1_ during follow-up (data not shown). Nrf2 target gene expression did not increase from baseline or differ among treatment groups in PBMC’s (Table 3).

Likewise, measures of other inflammatory or oxidative stress markers were not affected by sulforaphane treatment (Table 4). There were no differences among the groups in hematology or serum chemistry measures, lung function measures or patient reported dyspnea score or SGRQ scores, which reflect symptoms and activity limitations related to COPD at baseline or during follow-up (S1–S3 Tables, respectively).

The levels of the primary sulforaphane metabolites, the dithiocarbamates (DTC), in plasma were consistent with the treatment dose. DTC levels were not detectable or very low in the placebo group, higher in the 25 μmoles/day group, and highest in the 150 μmoles/day group (Fig 4).

Sulforaphane was well tolerated at both dose levels (S4 Table), although participants assigned to sulforaphane were more likely to report a bad taste (24% and 31% in the 25 and 150 μmoles dose groups respectively compared to 7% in the placebo group, P = 0.05). In addition, participants assigned to sulforaphane were more likely to report heartburn, nausea and abdominal discomfort, but the difference was not statistically significant (P-value less than <0.10 for all three). Two serious adverse events (hospitalizations due to cholecystitis and for a COPD exacerbation) were reported during the trial; both were in the low dose sulforaphane group and were not related to treatment.

## Discussion

There is a strong rationale from the published literature on animal models as well as human biospecimens from several diseases that Nrf2 is a key modifier of stress response against oxidative stress and inflammation [24,25]. For example, disruption of Nrf2 in mice model causes early onset and more severe emphysema after chronic cigarette smoke exposure [9]. Sulforaphane activates Nrf2 and turns on several antioxidant pathways [26] and its administration decreases oxidative stress and improves *in vitro* clearance of bacteria in macrophages from COPD patients [13]. In response to chronic cigarette smoke exposure, wild type mice fed with a potent activator of Nrf2, 1-[2-cyano-3-, 12-dioxooleana-1,9(11)-dien-28-oxy]imidazole (CDDO-Im), showed decreased oxidative stress and tissue damage compared to Nrf^-/-^mice [10]. Based on this rationale, we conducted a phase 2, randomized, placebo control trials to evaluate whether low or high daily doses of sulforaphane for four weeks stimulated Nrf2 target gene expression in patients with COPD. We did quality control testing of gel capsules stored at each of the three clinical centers over the course of the study that demonstrated that the sulforaphane content of the gel capsules was stable. Furthermore, sulforaphane has been shown to be bioavailable from gel caps and, similar to juice preparations, to be metabolized rapidly [20,27–29]. We confirmed that sulforaphane was well absorbed as evident from the levels of its dithiocarbamate metabolites in the plasma of participants. There was no effect of sulforaphane at either dose level on Nrf2 target gene expression in bronchial epithelial cells, alveolar macrophages or other cell types despite good adherence to the assigned treatment. In fact, target gene expression was not different from baseline in any of the treatment groups indicating there was no effect of sulforaphane. We also observed no effect on the markers of inflammation assessed, clinical measures of lung function or patient reported well-being.

Our understanding of the baseline level of Nrf2 in COPD patients remains unclear. Oxidative stress and inflammation are known to activate Nrf2, so there is a possibility that baseline level of Nrf2 activity is high in COPD patients. Hence, it would be difficult to further stimulate it with a small molecule activator such as sulforaphane, which could account for the lack of effect observed in this trial. However, in alveolar macrophages from COPD patients, sulforaphane did increase Nrf2 target gene expression and enhanced bacterial phagocytosis *in vitro* [13], which indicates a capacity for stimulation. Daily doses of sulforaphane for three or four days also were associated with enhanced phase 2 antioxidant enzyme expression in nasal cells from normal human volunteers [21], and altered responses to live-attenuated influenza vaccine (LAIV) [30] and diesel exhaust particles in nasal lavage cells [31], which indicates that oral doses can influence respiratory tract cells *in vivo*. However, similar to our study, Noah et al did not see effects on cytokine production or Nrf2 target gene expression in nasal epithelial cells after dosing subjects with sulforaphane [30]. It is unlikely that COPD patients suffer an irreversible loss of Nrf2 pathways that makes them resistant to Nrf2 stimulation since alveolar macrophages from COPD patients respond to sulforaphane *in vitro* [13]. Furthermore, our studies show that stratification of the groups as smokers and ex-smokers does not lead to any significant differences in Nrf2 activation by sulforaphane.

The doses of sulforaphane used in our study were comparable to or exceeded doses shown to stimulate anti-inflammatory pathways in other *in vivo* studies [21, 30, 31], however, the length of exposure in our study was longer than in those studies. Since we did not measure outcomes at earlier time points, we could have missed transient effects on the Nrf2 pathway. A randomized trial conducted in China to evaluate the effect of sulforaphane on detoxification of airborne pollutants showed an immediate beneficial effect of elevated urinary excretion of glutathione-derived conjugates of air pollutants that persisted throughout the 12 weeks of follow-up; urinary excretion of sulforaphane and its metabolites indicated rapid uptake and excretion of sulforaphane at the start and end of the 12 week period [27]. These results suggest that in individuals who are sensitive to the effects of sulforaphane, the effects are evident quickly and persist over time.

Strengths of our study include that is was designed to have sufficient power to compare a high and low dose to placebo and relied on outcomes shown to be sensitive to sulforaphane effects in prior studies. In addition, there were complete data collection on most participants and uniform procedures were used for processing of specimens collected at all three centers. Quality control tests included assessment of cell viability and measurement of RNA yields in each cell type overall and by clinical center; all of the results were within established norms. Moreover, all of the PCR was conducted on the same plates for baseline and follow-up samples and the outcome measures and laboratory analyses were performed blinded to treatment group. We also confirmed the absorption of sulforaphane in a dose-dependent manner by measurement of its dithiocarbamate metabolites in plasma.

Our study was the first randomized, placebo controlled clinical trial of sulforaphane supplementation in patients with COPD. We tested two doses of sulforaphane administered orally and there was no evidence of an effect on Nrf2 pathways at either dose level. Although our results are in conflict with other studies of sulforaphane effects, ours was the only study to evaluate the effects in lung tissues *in vivo* in patients with COPD. Tecifedra, also known as dimethyl fumerate or BG12, is the first Nrf2 activator that has been approved by FDA as a treatment for multiple sclerosis [32] and there are other molecules under development for multiple other diseases. It will be important to test inhalable formulation, which may be more efficacious than oral dosing, and other Nrf2 activators for COPD because a therapy targeting both oxidative stress and inflammation may be effective in modifying the disease such as COPD.

## Supporting Information

S1 Study Protocol (PDF)

S1 CONSORT Checklist (DOC)

S1 File Specimen Processing Methods. (PDF)

S1 Table Hematology and serum chemistry at baseline and change after 4 weeks by treatment assignment. (PDF)

S2 Table Pulmonary function measures, change from baseline at 4 weeks by treatment assignment. (PDF)

S3 Table Dyspnea and St. George Respiratory Questionnaire, change from baseline at 4 weeks by treatment assignment. (PDF)

S4 Table Treatment-emergent symptoms during follow-up by treatment group. (PDF)
