# Correction: Lack of Effect of Oral Sulforaphane Administration on Nrf2 Expression in COPD: A Randomized, Double-Blind, Placebo Controlled Trial

**DOI:** 10.1371/journal.pone.0175077

**Published:** 2017-03-28

**Authors:** Robert A. Wise, Janet T. Holbrook, Gerard Criner, Sanjay Sethi, Sobharani Rayapudi, Kuladeep R. Sudini, Elizabeth A. Sugar, Alyce Burke, Rajesh Thimmulappa, Anju Singh, Paul Talalay, Jed W. Fahey, Charles S. Berenson, Michael R. Jacobs, Shyam Biswal, on behalf of Broccoli Sprout Extract Trial Research Group

The following information is missing from the Funding section: Author SB is partly supported by the Flight Attendant Medical Research Institute Clinical Innovator Award.
