# Supplementary material for: Lack of Effect of Oral Sulforaphane Administration on Nrf2 Expression in COPD: A Randomized, Double-Blind, Placebo Controlled Trial
Source: PLoS One. 2016 Nov 10;11(11):e0163716. doi: 10.1371/journal.pone.0163716 (PMC5104323; doi:10.1371/journal.pone.0163716)
Supplement: S1 Study Protocol — (PDF) [file pone.0163716.s001.pdf]

National Institutes of Health

Broccoli Sprout Extracts Trial

(BEST)

U01 HL105569-01 (WISE and BISWAL)
IND 109233

Version 1.5
30 March 2012

Table of Contents

Abstract ...3
1. Introduction ...4
1.1 Title ...4
1.2 Sponsors ...4
1.3 Investigators and Study Centers ...4
1.4 Background and Significance ...4
1.5 Objectives ...7
2. Study design ...7
2.1 Hypotheses ...7
2.2 Trial Schema ...8
2.3 Study Treatment Groups ...8
2.4 Eligibility Criteria ...8
2.4.1 Inclusion Criteria: ...9
2.4.2 Exclusion criteria: ...9
2.5 Outcomes ...9
2.5.1 Primary outcome ...9
2.5.2 Secondary outcome ...9
2.6 Study Data Collection schedule ...10
2.7 Study Procedures ...11
2.7.1 Biospecimens ...12
2.7.2 Physiologic measures ...13
2.7.3 Patient reported outcomes ...13
2.7.4 Data and Specimen Storage and Transmission. ...14
3. Study Treatments ...14
3.1 Assigned treatments ...14
3.2 Drug Production and packaging / FDA Approval ...15
3.3 Side effects of study treatments ...15
3.4 Unmasking ...15
3.5 Routine COPD Treatment ...15
4 Statistical Design and Sample size ...16
4.1 Justification of Sample size ...16
4.2 General Statistical Approach ...16
4.3 Data and Safety Monitoring ...17
4.4 Study Organization ...17
4.5 Certification of Clinical Centers and Staff ...17
5. Protection of Human Subjects ...18
5.1 Risks to the Participants ...18
5.2 Steps taken to minimize the risks. ...19
5.3 Recruitment and Consent Procedures ...19
References ...21

Abstract

Chronic Obstructive Pulmonary Disease (COPD) is a major cause of morbidity and mortality in the United States and is a growing cause of chronic disease internationally. Presently, there are limited treatment options for this disease to modify the progression of airflow obstruction and decrease periodic exacerbations. Recent evidence has emphasized the central role of oxidative stress as a mechanism of COPD pathobiology. Evidence from our group has shown that COPD patients and animals exposed to cigarette smoke have impairment of antioxidant defenses which are caused by a defect in activity of Nrf2, a prolific regulator of anti-oxidant enzymes, glutathione homeostasis, and cytoprotective proteins. Activation of Nrf2 protects mice with chronic smoke exposure from developing emphysema, decreases oxidative stress, increases proteasomal anti-apoptotic cytoprotective responses, improves bacterial phagocytosis and killing, and reverses tobacco-smoke induced corticosteroid resistance. Similarly, *in vitro* Nrf2 activation in human COPD lung cells has shown improved cytoprotection, improved bacterial clearance, and restoration of steroid sensitivity. This trial focuses on sulforaphane, a derivative of cruciferous vegetables, which is a potent *in vitro* and *in vivo* stimulator of Nrf2 activity. We want to investigate whether ingestion of sulforaphane by COPD patients will increase Nrf2 activity and expression of downstream antioxidants in alveolar macrophages and bronchial epithelial cells. Accordingly, we are conducting a placebo-controlled randomized proof of principle trial of two oral doses of sulforaphane, 25 and 150 micromoles, for 4 weeks in 90 COPD patients. Collections of alveolar macrophages by BAL, bronchial epithelial cells by endobronchial brushings will be performed at baseline and 4 weeks. Other biospecimens will include nasal epithelial cells, Peripheral Blood Monocyte Collection (PBMCs), and expired breath condensate (EBC). Our goal is to establish a safe and tolerable dose of sulforaphane that effects *in vivo* antioxidants via Nrf2, then we will have a novel candidate treatment for longer-term efficacy trials. Ancillary studies are proposed to explore the efficacy and mechanisms of sulforaphane to increase bacterial clearance and to restore steroid sensitivity in COPD lung cells.

1. Introduction

1.1 Title

Broccoli Sprout Extracts Trial (BEST)

1.2 Sponsors

National Institutes of Health/National Heart Lung Blood Institute U01 HL105569-01 (Wise and Biswal)

1.3 Investigators and Study Centers

Participating Clinical Centers

Wise, Robert	Johns Hopkins School of Medicine
Criner, Gerard	Temple University
Sethi, Sanjay	University at Buffalo, The State University of New York

Data Coordinating Center

Holbrook, Janet	Johns Hopkins Bloomberg School of Public Health

Biospecimen Analysis Laboratory

Shyam Biswal	Johns Hopkins Bloomberg School of Public Health

Broccoli Sprout Extract Production and Analysis Center

Jed Fahey	Johns Hopkins University School of Medicine
Paul Talalay	Johns Hopkins University School of Medicine

1.4 Background and Significance

Chronic Obstructive Pulmonary Disease (COPD) is a highly morbid disease, mainly caused by cigarette smoking in the United States, afflicting 24 million people. More than 125,000 people die from COPD each year, making it the fourth leading cause of death in the U.S. The total costs for COPD care are estimated to be $29 billion per year.[1]

Current Treatments for COPD

Despite the rising morbidity and mortality, there are few new treatments that can arrest the progress of the disease. Drug treatment relies largely on bronchodilators and inhaled corticosteroids, which improve lung function and reduce exacerbations but have limited effect on progression of the disease. Aside from smoking cessation, there are no treatments that directly address the underlying pathobiology of COPD.

Role of bacterial exacerbations in COPD

Patients with COPD have, on average, 1-3 exacerbations per year, but the frequency varies widely among patients. Exacerbations are associated with faster loss of lung function, impaired quality of life, increased mortality, and greater costs of healthcare.[2,3] An estimated 10% of patients with frequent and severe exacerbations account for 70% of the total health care use.[4] Risk factors include the presence of cough and mucus, and colonization of the lower airways by bacteria. Approximately 50% of exacerbations are due to bacterial infections, most frequently non-typeable H. Influenza. A major goal of COPD treatment is to reduce exacerbations.

Disease Modification in COPD

Although treatment moderately reduces the frequency of exacerbations and improves lung function, there are no treatments that clearly alter the progression of the disease through interruption of the chronic inflammatory processes that lead to parenchymal destruction and airway remodeling. Thus, there is a need for new approaches to therapy that can address directly the pathobiologic alterations that occur in COPD.

Pathobiology of COPD

Only a minority of smokers develop clinically important COPD. In recent years, our understanding of the pathobiology of COPD has increased considerably. Among other key findings, we now know that patients with COPD, in contrast to smokers without COPD have the following characteristics.

- Increased burden of oxidative stress and impaired anti-oxidant defenses.[5 6 7]
- Impaired macrophage function including phagocytosis and bacterial killing.[8 9]
- Resistance to the anti-inflammatory effects of corticosteroids.[10]
- Persistent pulmonary and systemic inflammatory response. [11 12]

These phenomena account for some of the clinical characteristics of COPD including:

- the progression of disease in some individuals after smoking cessation [13]
- the frequency of bacterial infections causing exacerbations of disease[14]
- the relative ineffectiveness of inhaled steroids in COPD compared to asthma[15]

Oxidative stress in COPD

There is a growing body of evidence that oxidative stress plays a pivotal role in the development and maintenance of the inflammatory response in COPD. Increased oxidative stress has been found in biological samples from the bronchial lavage, expired breath, blood, and urine in patients with COPD compared to smokers without COPD and non-smokers.5 6 7 [16] The consequences of oxidative stress include: 1) inactivation of antiproteases, 2) down regulation of HDAC2 causing steroid insensitivity, [10] 3) alveolar epithelial injury and apoptosis 4) gene expression of inflammatory mediators.5 7 4) Oxidative stress may stimulate mucus secretion and impair mucociliary clearance. [17] 5) Decrease alveolar macrophage bacterial killing. [18] Accordingly, it is logical to target oxidative stress as a promoter of the clinical manifestations of COPD including parenchymal destruction of the lung and recurrent exacerbations.

Failure of Nrf2 – a unifying pathway to COPD

Biswal's laboratory has focused on the role of *Nuclear factor erythroid 2-related factor 2* (Nrf2) in the pathogenesis of COPD. Nrf2 is a prolific transcription factor, induced by oxidative stress, that binds to the antioxidant response element (ARE) and induces a broad program of antioxidant enzymes, glutathione homeostasis, and detoxification enzymes including anti-proteases.[19 20 21] Moreover, we have recently demonstrated that COPD patients are deficient in Nrf2. [22]

Preliminary Data

Key preliminary findings from the Biswal laboratory have shown the following:

- In tobacco-exposed mice, Nrf2 protects from cigarette smoke induced oxidative injury and emphysema.[23]
- In tobacco-exposed mice, Nrf2 activation by genetic disruption of Keap1 protects from cigarette-smoke induced lung injury [24]
- In tobacco exposed mice, pharmacological activation of Nrf2 by sulforaphane and other small molecules protects from CS-induced oxidative lung injury and emphysema [25]

- In lung tissues from humans with COPD, decline in Nrf2 and Nrf2-regulated cytoprotective defenses in lungs correlates with severity of COPD [22]
- In mouse lungs , in-vivo treatment with sulforaphane upregulates Nrf2-directed cytoprotective defenses.[25]
- In human bronchial epithelial cell lines, in-vitro treatment with sulforaphane upregulates Nrf2-directed antioxidant and cytoprotective defenses.[26]
- Sulforaphane treatment inhibits bacterial colonization in CS exposed Nrf2+/+ mice but not in Nrf2-/- knockout mice (preliminary data)
- Sulforaphane treatment in-vitro improves bacterial phagocytosis in COPD alveolar macrophages via Nrf2 mediated increase in surface receptor MARCO (Preliminary data)
- Sulforaphane treatment in-vitro improves corticosteroid protection in COPD alveolar macrophages exposed to LPS (preliminary data)

Thus, it appears that reduction in Nrf2 can account for many of the observed defects in COPD, and that treatments that stimulate the production of Nrf2 is a logical approach to treatment of the disease with a goal of disease modification – slowing of parenchymal destruction and resistance to bacterial exacerbations.

<u>Antioxidant strategies for COPD</u> Trials of supplemental antioxidants using NAC and vitamin E show modest or no effect on FEV1 decline.[27] [28] [29] This failure may be attributed to the fact that these single antioxidant molecules gave insufficient protection at the lung tissue level or may have been given in insufficient dose to affect susceptible tissues. On the other hand, a novel strategy based on targeting Nrf2, which up-regulates a broad range of cytoprotective genes including antioxidants is a much more promising therapy as evidenced in the preclinical data.[30]

<u>Nrf2 Activators</u> Several compounds are effective in increasing Nrf2 in preclinical models. These include dithiolethiones [31], sulforaphane[32], the triterpenoid CDDO[33], reseveratrol[34], curcumin[35], and quercitin [36]. Among these sulforaphane is the most extensively studied in humans and has the most relevant pre-clinical support for use in COPD. Sulforaphane is an isothiocyanate that is derived from mastication of cruciferous vegetables which converts the glucosinolate form to the active compound. Sulforaphane has several demonstrated properties that suggest that it would be an ideal treatment for COPD.

- It stimulates Nrf2 and downstream anti-oxidants, proteasomal subunits and other cytoprotective genes in lung tissue [37]
- It reduces ER stress that leads to apoptotic cell death in tobacco exposed lungs[38]
- It confers increased activity to macrophages to phagocytose and kill bacteria that cause COPD exacerbations in tobacco exposed mouse lungs and human COPD macrophages. (see ancillary study 1)
- It reduces oxidative / nitrosative modifications of critical proteins such as HDAC2 that mediate corticosteroid anti-inflammatory resistance in COPD (see ancillary study 2)

<u>Other biological effects of sulforaphane</u>
In pre-clinical studies, sulforaphane has a number of other potentially beneficial effects that may confer benefit to COPD patients with respect to co-morbid conditions such as lung cancer, arteriosclerosis, and respiratory viral infections.

- Sulforaphane suppresses endotoxin-induced inflammatory response by inhibiting TLR4 and NF-KB signaling[39]
- Sulforaphane inhibits lung RSV replication and virus-induced inflammation in mice[40]

- Sulforaphane suppresses inflammatory responses in endothelial cells and arterial damage induced by shear stress[41]
- Sulforaphane suppresses T-cell activation and protects from arthritis in mice [42]
- Sulforaphane protects from ischemic injury of the heart through oxidative stress[43]
- Dietary sulforaphane protects against hypertension and atherosclerosis by reducing oxidative stress[44]
- Sulforaphane confers neuroprotection in endotoxin, cerebral hemorrhage and trauma by suppressing inflammation and oxidative stress. [45] [46]
- Sulforaphane protects against inflammatory skin disorders including UV induced skin injury [47] and epidermolysis bullosa [48]
- Sulforaphane exhibits anti-bacterial activity against Helicobacter pylori and prevents benzopyrene-induced stomach tumors[49]
- Sulforaphane inhibits malignant progression of lung adenomas induced by tobacco carcinogen in mice[50] as well as anticancer properties in other organs[51]

Potential Impact of the Proposed Research

If we are able to demonstrate that orally administer sulforaphane has similar biological properties as it has *in-vivo* in experimental animals exposed to tobacco and *in-vitro* to human COPD lung cells, this will provide us with an agent that can be readily developed to be taken into Phase III trials. It would suggest that sulforaphane would be effective not only in limiting the progression of COPD, but also decrease the frequency and severity of exacerbations. The clinical importance of developing a drug that can effectively modify the progression of COPD would not only alleviate suffering and reduce healthcare expenditures, but would address what is arguably the most significant unmet need in pulmonary medicine in the world. We are also optimistic that sulforaphane has potential for distribution not only as a drug, but also in the form of nutritional supplementation or dietary modification which would make it accessible to vast numbers of individuals in third-world countries where COPD is a growing problem.[52]

1.5 Objectives

The primary goals of this trial are to:

- Establish proof of principle -- Does oral sulforaphane increase Nrf2 expression and downstream anti-oxidant enzymes in lung macrophages and epithelial cells.
- Determine tolerability and dose-ranging for subsequent clinical research
- Establish non-invasive surrogate biomarkers for Nrf2 expression in the lung
- Conduct ancillary mechanistic studies to determine whether *in-vivo* treatment increases bacterial phagocytosis and killing and decreases corticosteroid resistance

2. Study design

2.1 Hypotheses

Daily ingestion of 25 or 150 micromoles of sulforaphane for 4 weeks will have the following dose-dependent effects:

H1. Increase Nrf2 activation in alveolar macrophages and bronchial epithelial cells

H2. Increase phase II anti-oxidant enzymes in alveolar macrophages and bronchial epithelial cells

H3. Reduce markers of oxidative stress in expired breath condensate

H4. Increase bacterial clearance of clinical isolates of Hemophilus influenza and Pseudomonas aeruginosa from COPD patient by alveolar macrophages. (Ancillary Study 1)

H5. Increase steroid (dexamethasone) sensitivity to suppression of IL8 release by LPS in alveolar macrophages. (Ancillary Study 2)

Exploratory studies will be done to determine whether sulforaphane has lung anti-inflammatory properties and whether non-invasively collected specimens (blood and nasal brushings) can be used as surrogates for response of lung cells.

2.2 Trial Schema

The trial will be a 3-arm parallel design randomized placebo-controlled trial in 90 COPD patients. After screening (V1), participants are scheduled for a baseline clinical evaluation (V2) and a bronchoscopy (V3) within a week. The clinical evaluation (V4) and bronchoscopy (V5) are repeated 4 weeks later. Although the effects of sulforaphane on nasal epithelium are known to occur within several days, the 4 week treatment period is long enough to permit theinflammatory response from the first procedure to subside and to provide an assessment of tolerability of the treatment.

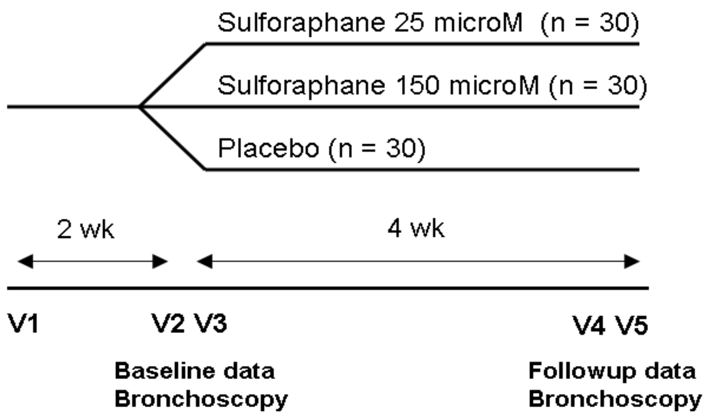

2.3 Study Treatment Groups

There will be 3 parallel treatment groups:

- Sulphoraphane capsules 4.4 mg (25 micromoles) once daily by mouth
- Sulphoraphane capsules 26.6 mg (150 micromoles) once daily by mouth
- Matching Placebo (microcellulose) once daily by mouth

2.4 Eligibility Criteria

The entry criteria are designed to select patients who meet GOLD criteria for COPD who are likely to tolerate repeated bronchoscopies. Both active and former smokers will be enrolled because we are uncertain whether this will influence the outcome. Both active and former smokers show Nrf2 deficiency and in-vitro response to Nrf2 promoters. Patients are asked to limit intake of cruciferous vegetables (e.g. broccoli, cabbage, collard / mustard greens, kale, brussels sprouts, radishes) to no more than 1 serving per week to avoid confounding of results by dietary intake of sulforaphane.

2.4.1 Inclusion Criteria:

1. Age 40 or greater, either sex
2. 10 or more pack-years smoking history
3. Physician diagnosed COPD
4. Post bronchodilator FEV1/FVC ratio < 0.70
5. FEV1 40-80 % predicted
6. Willingness to ingest no more than 1 serving of cruciferous vegetables per week during run-in and treatment periods
7. Ability and willingness to provide informed consent

2.4.2 Exclusion criteria:

1. COPD exacerbation within preceding 6 weeks requiring treatment
2. Significant respiratory (other than COPD), cardiovascular, neuropsychiatric, renal, gastrointestinal, or genitourinary disease that would interfere with participation in the study or interpretation of the results.
3. Acute MI or Acute Coronary syndrome within 6 prior months
4. Cancer (other than skin or localized prostate) within preceding 5 years
5. Child-bearing potential with lack of adequate contraception, Pregnancy or lactation. Acceptable forms of birth control include abstinence, hysterectomy, tubal ligation, two of the following: vasectomy, condom, diaphragm, intrauterine device, oral or implanted contraceptives, or spermicide.
6. Allergy to local anesthesia
7. Resting hypoxemia (O_2 saturation <90%)
8. Glomerular Filtration Rate (GFR) < 30 ml/min
9. Liver enzymes four times upper normal
10. Current use of warfarin for any indication

2.5 Outcomes

Since this is a proof of principle study, we do not have a single primary outcome, but are evaluating several markers of Nrf2 activation and anti-oxidant activity. We will consider that we have established proof of principle if we can demonstrate an increase in NrF2 expression and increased expression of at least two anti-oxidant enzymes.

2.5.1 Primary outcome

1. <u>Nrf2 levels and downstream anti-oxidants</u> The primary design variables are the change in Nrf2 expression in alveolar macrophages (AM) and bronchial epithelial cells (BEC). Support of antioxidant effects will be quantified by changes in phase II anti-oxidant gene expression (NQ01, HO-1, GPX2, GCLM, GCLC, GSTA1). We will correlate changes in these levels with non-invasive measures from peripheral blood monocytes and nasal epithelial cells.

2.5.2 Secondary outcome

Supportive secondary outcome measures will include measures of oxidant stress from plasma and expired breath condensate. We will also conduct ancillary exploratory studies to determine whether the treatment improves function of alveolar macrophages in bacterial clearance and improves steroid sensitivity. Markers of oxidative stress include 8-isoprostane, H2O2, GSH/GSSG, Nitrotyrosine, Protein carbonyls, TBARS

1. Measures of oxidative stress. Oxidant stress indicators (isoprostane) will be measured in plasma and expired breath
2. Measures of airway inflammation will be measured as BAL cell counts and cytokine profiles
3. Pulmonary function tests including spirometry will be measured to determine whether there are any short-term functional effects of the treatment.
4. Patient Reported Outcomes will include the MRC dyspnea scale and SGRQ which includes questions on cough and sputum production.[53] [54] Adverse events will include both spontaneous reports as well as targeted symptoms related to respiratory and GI systems. Adverse events will be scored based on the NCI Common Toxicity Criteria rating scale.
5. Safety Measures will include baseline (V1) and follow-up (V4) CBC and biochemical profile including renal function, thyroid function, and liver function tests. Women of childbearing potential will have a pregnancy test at V1 and V4.

2.6 Study Data Collection schedule

The specific study procedures and approximate time commitment from the participant are shown below:

Participants will have 5 study visits over 4-8 weeks as follows:

Visit 1 (V1) (Day – 28 to -7) (2 hours) During this visit, the participant will provide informed consent, undergo eligibility screening questionnaire, lung function (spirometry before and after bronchodilators), Complete Blood Count, biochemistry panel including renal and liver function and electrolytes, TSH, urinalysis, and a pregnancy test for women of child-bearing potential.

Visit 2 (V2) (Day -14 to 0) (2-4 hours) During this visit, eligible participants will perform baseline spirometry, lung volumes, and diffusing capacity, and will complete general health questionnaires and respiratory specific questionnaires (ATS-DLD, SGRQ and MRC). A brief physical examination will be performed. Baseline biological specimens including blood for PBMCs will be performed.

Visit 3 (V3) (Day 0) (4 hours) The participant will have a baseline bronchoscopy for collection of bronchoalveolar lavage cells and nasal and bronchial epithelial brushings. Randomization eligibility will be established and the participant will be assigned to a masked treatment. After recovery from the bronchoscopy sedation, the participant will be given the assigned treatment which will consist of a coded container of capsules containing either placebo or one of the two doses of sulforaphane.

Phone Visit 1 (P1) (Day 1) (15-30 minutes) Patients are phoned the day following the bronchoscopy to determine whether they have had any adverse experiences, to re-instruct them in use of the medication, and to confirm future appointment dates and times.

Phone Visit 2 (P2) (Day 7-14) (15-30 minutes) Patients are contacted to determine their compliance with the medication and to report any adverse effects.

Visit 4 (V4) (Day 21 to 30) (2 – 4 hours) The participant will repeat the baseline spirometry, lung volumes and diffusion capacity, CBC, pregnancy test, urinalysis, TSH and biochemistry panel and have follow-up health status and adverse effect questionnaires and a brief physical examination. Follow-up collections of blood for biological measures are obtained.

Visit 5 (V5) (Day 22 to 31) (4 hours) Follow-up bronchoscopy for collection of bronchoalveolar lavage, nasal brushings, specimens and endobronchial brushings. Return of unused capsules for pill counts.

Phone Visit 3 (P3) (Day 24 to 38) (15-30 minutes) Exit interview and followup for adverse effects of bronchoscopy.

Table 1: Study Data Collection Schedule

Visit	V1	V2	V3	P1	P2	V4	V5	P3
Time window (days)	-28 to -7	-14 to 0	0	1	7-14	21-30	22-31	24-38
Target (days)	-21	-14	0	1	14	25	26	27
Consent, eligibility evaluation	•							
Screening Questionnaire	•							
Spirometry/PFTs	•	•				•		
Pulse Oximetry	•	•						
Lung volume/DCO		•				•		
CBC, Chemistry panel, TSH	•					•		
Urine analysis	•					•		
Physical Exam		•				•		
Pregnancy Test	•					•		
Randomization			•					
Health Status / AE		•				•		
SGRQ/MRC		•				•		
ATS-DLD		•						
EBC		•				•		
PBMC/Plasma/Serum		•				•		
Nasal Brush			•				•	
BAL/Bronchial Brush			•				•	
Adherence Counseling				•	•			
Exit interview								•

KEY: AE = adverse event questionnaire, ATS-DLD = ATS-DLD respiratory questionnaire, BAL = Bronchoalveolar lavage, CBC = complete blood count, DCO= Diffusion capacity for carbon monoxide, EBC = Expired Breath Condensate, MRC = Medical Research Council Dyspnea Scale, PBMC = Peripheral Blood Monocyte Collection, SGRQ = St Geroge's Respiratory Questionnaire, TSH = Thyroid Stimulating Hormone, UA = Urinalysis

2.7 Study Procedures

A variety of biological samples will be collected from patients with COPD for analysis of Nrf2 activity and related phase II enzymes.

2.7.1 Biospecimens

Bronchoalveolar lavage (BAL). Patients, fasting for 6 hours prior to the procedure, are sedated with midazolam and fentanyl. The bronchoscope is introduced via the nares into the trachea under local anesthesia with 1% or 2% lidocaine. The bronchoscope is wedged into two subsegments of the right lung (anterior segment of upper lobe, and lateral segment of middle lobe). In each subsegment 3 x 50 ml aliquots of normal saline are instilled and withdrawn. The lavage material, which averages 25% return in patients with COPD contains typically yields 1-10 x 10^6 macrophages which are sufficient for all of the assays proposed in this study. The centrifuged cell pellet is divided into two aliquots and one aliquot is stored at -70C. The supernatant is divided into 4-5 aliquots and stored in cryotubes.

Bronchial brushings. Prior to the bronchoalveolar lavage, a cytology brush is inserted into the bronchoscope channel and brushings are collected from 4 quadrants of visible subsegments of the right lung under direct visualization. The cellular material is washed off in saline and the procedure is repeated 10 times. The liquid is centrifuged and the cell pellet is stored at -70C.

Nasal brushings. Each nostril is anesthetized with lidocaine 1% spray. A nasal cytology brush is gently rubbed along the lower turbinate and rinsed in normal saline. The procedure is repeated three times in each nostril. The centrifuged cell pellet is stored at -70C.

Peripheral Blood Monocytes Collection (PBMC). 60 ml of blood is withdrawn by venipuncture and the buffy coat is separated. Monocytes are separated in a Ficoll-Hypaque gradient, washed and frozen at -70C.

Plasma. Plasma from the blood collection is prepared by centrifugation and stored at -70C. This plasma will be held for exploratory supplementary measures of cytokines that are not included in this application.l

Expired Breath Condensate (EBC) is collected using the R-tube method in which a refrigerated aluminum tube is placed over a disposable valved collecting tube and condensate trap. After 5-10 minutes of quiet tidal breathing, approximately 0.5-1.0 ml of condensate is collected and frozen at -70C pending measurement of isoprostane.

Blood draw 10-20 ml of blood is collected by venipuncture at V1 and V4 for complete blood count and biochemistry profile (comprehensive metabolic panel). These tubes are transmitted to the local hospital laboratory without processing.

Urine. A spot sample of urine will be collected and analyzed for routine urinalysis in the local hospital laboratory

Table 2: Biological measures to be assessed in different biological samples

Procedure	Biological sample	Biological measures*
Exhaled breath condensate (EBC) collection	EBC	Oxidative stress markers*
Blood collection	PBMC	Nrf2 activity**
	Plasma	Oxidative stress markers
Nasal brushing	Nasal epithelial cells	Nrf2 activity
Bronchoscopy		
• Bronchial brushings	Bronchial epithelial cells	Nrf2 activity, oxidative stress markers
• Bronchoalveolar lavage (BAL)	Alveolar macrophages	Nrf2 activity, inflammatory cytokines***, NF-KB
	BAL fluid	Inflammatory cytokines

*Oxidative stress markers include Isoprostane and, in fresh specimens H2O2.
**Nrf2 activity: Nrf2 activity will be assessed by measuring: 1) Nrf2 protein, 2) expression of a panel of Nrf2 regulated genes (antioxidants and proteasomal subunits) 3) GSH levels and 4) Proteasomal activity.
***Cytokine assays include IL6, IL8, TNF-alpha, INF-gamma, and MIP.

2.7.2 Physiologic measures

Spirometry

Spirometry will be performed at V1, V2, and V4 in accordance with ATS/ERS recommendations. Spirometry reference values will be those of Hankinson et al from NHANES. At V2, and V4 participants will also have lung volume measurements and diffusing capacity measurements performed for the purpose of characterization and to exclude patients with restrictive lung disease. These measures will be performed in accordance with local hospital laboratory procedures.

2.7.3 Patient reported outcomes

Questionnaires

ATS-DLD respiratory questionnaire as modified for use in the Lung Tissue Research Consortium (LTRC) will be completed at V2. This instrument is used to define respiratory exposures to cigarettes and other occupational / environmental agents as well as to describe severity and frequency of respiratory symptoms such as cough, phlegm, and dyspnea.

St. George's Respiratory Questionnaire (SGRQ) will be completed at V2 and V4. This is a validated self-completed questionnaire that assesses respiratory-specific quality of life and disease impact with 3 domains and an overall score. We are not expecting to find significant short-term changes in SGRQ with sulforaphane treatment, however we are using this instrument to characterize the disease severity of our treatment population and acquire measures of short-term intra-individual variability that will assist us with planning of future trials should this approach be used in the future.

Medical Research Council (MRC) dyspnea scale will be completed at V2 and V4 to categorize the extent of activity limitation due to dyspnea on a 5 point scale.

Health Status and Adverse Event questionnaires will be completed at V2, P2, and V4 to ascertain whether there have been any significant illnesses, interval illnesses or changes in treatment, or new symptoms reflecting adverse events. Typically targeted adverse events are asked about specifically, and other adverse events are solicited by open-ended questioning and categorized as mild, moderate or severe using standard definitions. Because sulforaphane has such a clean profile with respect to adverse events, we are not planning to ask about specific targeted sulforaphane events, but we will ask about interval respiratory infections or exacerbations as well as specific symptoms related to the bronchoscopy procedures.

2.7.4 Data and Specimen Storage and Transmission.

The primary analysis for Nrf2 protein, mRNA, and antioxidant enzymes in cell pellets will be conducted in the laboratory of Dr. Shyam Biswal at the Johns Hopkins University School of Public Health. Initial processing of the specimens will take place at each of the clinical sites. Cell pellets from bronchoalveolar lavage, endobronchial brushings, peripheral blood monocytes, and nasal epithelial cells will be frozen at -70C and shipped on dry ice in batches to the Biswal laboratory for analysis. All of the laboratory methods have been well established in the Biswal laboratory, and Nrf2 protein and message as well as antioxidant enzymes have been stable in frozen specimens.

The ancillary studies will be done with fresh alveolar macrophages and thus will be done on a subset of specimens. Measurement of bacterial phagocytosis and killing will be done at Dr. Sethi's laboratory at SUNY Buffalo and at Dr. Biswal's laboratory at Hopkins (n = 120 samples (60 pairs)). Measurement of IL8 production by LPS stimulated macrophages and suppression by dexamethasone will be conducted on fresh specimens from Hopkins (n = 60 samples (30 pairs)).

Data, which includes identifiable personal health information (PHI), are collected at each of the clinical sites. PHI is stored at each of the clinical sites in accordance with HIPAA regulations and local university and hospital policies. This includes the storage of PHI in locked cabinets or rooms, limited access to secure data areas by certified participating study personnel, password protection for electronic medical records, and explanation of HIPAA regulations on the study consent form. Data such as lung function or laboratory tests that is collected as part of this study may be transmitted to the patients treating physicians with the consent of the participant. Participants are informed in the consent that PHI may also be disclosed for auditing purposes by the FDA or other regulatory bodies and is subject to subpoena.

PHI is not transmitted to the coordinating center or central laboratory in that all specimens and records are identified by an anonymous study ID, and other identifying information such as name or hospital ID number are not entered into the central study database. Source records that are transmitted to the coordinating center for data quality audits have identifying information redacted.

3. Study Treatments

3.1 Assigned treatments

1. 25 micromoles (4.4 mg) sulforaphane once daily by mouth
2. 150 micromoles (26.6 mg) sulforaphane once daily by mouth
3. Placebo (microcellulose) once daily by mouth

3.2 Drug Production and packaging / FDA Approval

Sulforaphane will be prepared under the direction of Drs. Talalay and Fahey in accordance with GMP using procedures that have been well described.[55] Briefly, the procedure, performed in an FDA-certified facility, uses a hot-water pulverization and extraction of glucosinolates from standardized patented broccoli sprouts. (Broccosprouts®, Tracy, CA) The extract is treated with myrosinase from daikon sprouts to form the isothiocyanate. The homogenate is lyophilized and pulverized, and stored as a frozen powder. After analysis of sulforaphane concentration by cyclocondensation,[56] the material is encapsulated at the two dose levels at a GMP manufacturing pharmacy and backfilled with methylcellulose. Similar capsules are filled with methylcellulose alone to serve as placebos. Study drug is stored at -20 C under which conditions the drug is known to be stable for at least one year until it is dispensed and thereafter is maintained in a refrigerator for the 4 week duration of the study. Because the material is hygroscopic, we are recommending that it be stored in a refrigerator during the 4 weeks of the trial, although we are not certain that this is an absolute requirement. The trial will be conducted under an IND (#109233) held by Robert Wise, MD

Participants will be provided with a one month supply of treatment at randomization. Distribution, auditing and maintenance of treatment inventories at the clinical centers including monitoring of expiration dates will be managed as a feature of the data system.

3.3 Side effects of study treatments

Ingestion of Broccoli Sprout Extract (BSE) containing sulforaphane is thought to be of minimal risk to individuals given the fact that broccoli and broccoli sprouts are widely consumed throughout the world and are without known adverse effects. Sulforaphane in the precursor glucosinolate form is widely available as a nutritional supplement in health food stores. However, the intestinal conversion to sulforaphane in the isothiocyanate form may be variable, depending on the intestinal flora, so we have elected to use the highly purified and quantified form for this trial. To date, there are no known significant adverse effects related to ingestion of the substance.

3.4 Unmasking

Drug and placebo will be supplied in similar appearing capsules, back-filled with methylcellulose to approximate similar appearance and weight. Neither the participant nor the site investigators or the laboratory personnel will know the identity of the drug assignment. Emergency unmasking can take place by opening a sealed envelope that is distributed with the drugs. All unmasking events are reported to the DCC. The sealed envelopes are returned to the DCC at the end of the trial. Also, the investigators doing the bioassays will do them masked to treatment assignment. It is our general position that unmasking of treatment assignment is rarely necessary. If adverse events occur that may be related to the study treatment, then the study treatment is stopped and the patient is followed on an intention to treat basis. Envelopes with treatment assignment are provided to the clinic in the case a treating physician feels it is important to know the treatment assignment.

3.5 Routine COPD Treatment

All participants will be able to continue with their routine COPD care which largely consists of long-acting bronchodilators and inhaled corticosteroids. The only change that we are asking our participants is to limit their intake of cruciferous vegetables to no more than one serving per week. Based on food-frequency information in COPD patients, this should not represent an

undue burden and will be easy for most to adhere to. Participants will be given a flashcard listing cruciferous vegetables.

4 Statistical Design and Sample size

4.1 Justification of Sample size

Power Analysis: For a one-way ANOVA, assuming 30 samples per each of 3 groups, and 80% power, the detectable effect size by Cohen's *f* statistic is 0.333, which corresponds to a medium effect size. The sample size is based on SD estimated from assays of human COPD lung tissues and nasal epithelial cells.[22] The detectable fold-difference for primary and secondary analytes is listed in the table below. Estimates are also provided for a nominal type I error of 0.01 to account for multiple comparisons.

	Estimated SD (Fold-change)	Effect Size (3 groups of n = 30, power = 0.80)	
Analyte		alpha = 0.05	alpha = 0.01
Nrf2 protein	0.198	0.066	0.080
Nrf2 mRNA	1.007	0.335	0.406
GTSM	0.397	0.132	0.160
GSTP1	0.273	0.091	0.110
HO-1	0.427	0.142	0.172
NQ01	0.484	0.161	0.195

These effect sizes are highly plausible and have potential clinical importance insofar as they compare to changes of 1.25-4.00-fold in Nrf2 activity in control smokers vs. patients with COPD, and 2-3-fold changes in anti-oxidant enzymes induced by oral sulforaphane in control nasal epithelium. Tests for trend for these analytes vs. dose with 90 patients in 3 groups has 80% power with type I error of 0.05 to detect a correlation coefficient of 0.26 or greater.

4.2 General Statistical Approach

Primary analysis. Baseline variables and outcome measures will be examined using descriptive and graphical displays such as boxplots and scatter plots. Appropriate transformations will be applied to data that is non-parametric. The primary analysis will be an unadjusted one-way ANOVA for fold-change in Nrf2 expression and phase II antioxidant levels with sulforaphane dose as the main effect. Post-hoc comparison between doses will use Tukey's test. Dose-response relationships will be tested with a test for trend (Pearson's r). If significant imbalance occurs between groups, then general linear models will be constructed to adjust for baseline differences. The primary analysis will use the modified intention to treat paradigm.

Secondary analyses. Secondary outcome measures will be similarly analyzed, but p-values will be adjusted for multiple comparisons. Subgroups of interest (gender, smoking status, GOLD stage, adherent participants) will be examined by tests of treatment by subgroup interaction.

Early stopping rules. We are not proposing any interim efficacy analysis or early stopping rules.

Participants will continue to be enrolled in the trial regardless of whether they take the study medication, as our first analysis will be an intention to treat analysis. Secondary analyses will

include only adherent patients. Patients may be removed from the study if they sustain interim adverse events related to the study, if they are not healthy enough to undertake the study procedures (e.g. bronchoscopy).

4.3 Data and Safety Monitoring

An independent Data and Safety Monitoring Board (DSMB) approved by NHLBI will be appointed for approving the protocol for the BEST trial and monitoring the trial progress.
The DSMB will meet twice a year, usually by teleconference, to review data from BEST. The DSMB is also responsible for review of related issues, such as center performance standards or recruitment incentives. The DSMB may request more frequent meetings if necessary to fulfill its charge. It may also request additional safety reports on a more frequent basis. After each meeting, the DSMB will make formal recommendations regarding trial continuation and clinical performance. The DSMB recommendations will be submitted to participating center's IRB's. A special responsibility of the DSMB is to review serious adverse events (SAEs), as defined by deaths, life threatening conditions, hospital admissions, adverse pregnancy outcomes or events requiring permanent discontinuation of the treatment. SAEs are to be reported to the DCC within 72 hours of notice, with follow up reporting until the event has terminated. SAEs will be reported to the Chairman of the DSMB or other designee in a timely fashion for review. This initial review will determine whether there is a recommendation for a change in the protocol or halt to the study until such time as the matter can be considered by a quorum of the DSMB. In addition, the DCC will distribute reports of SAEs from one center to all of the centers for review by the local IRBs.

4.4 Study Organization

The study will be co-directed by Drs. Wise and Biswal who will serve as the joint Principal Investigators. Dr. Wise will direct the clinical research aspects of the study and Dr. Biswal will direct the laboratory-based aspects of the study. Dr. Talalay, with Dr. Fahey will supervise the extraction, purification, analysis, and packaging of the sulforaphane. Dr. Holbrook will direct the data coordinating center and the acquisition and analysis of the data. Three clinical sites, each of which will be recruiting 30 patients, will be supervised at SUNY Buffalo by Dr. Sethi, at Temple by Dr. Criner, and at Hopkins by Dr. Wise. The overall scientific direction of the study will be directed by the Scientific Steering Committee consisting of Drs. Biswal, Criner, Holbrook, Sethi, Talalay, and Wise. The two ancillary studies will be supervised by Dr. Sethi (Sulforaphane Effect on Bacterial Phagocytosis) and Dr. Thimmulappa (Sulforaphane Effect on Steroid Sensitivity).

4.5 Certification of Clinical Centers and Staff

Clinical centers must be certified by the DCC prior to enrolling patients into the trial and clinical staff must be certified before they can participate in data collection or data entry.

- Clinical certification requirements
  <u>Training:</u> Attendance by the clinical coordinator at a training meeting to discuss study background and rationale, methods and procedures, research ethics, data collection and entry, and principles of good clinical practice. Clinics can receive provisional certification through a private training session with a study monitor before the training meeting.

  <u>Facilities:</u> Clinics must demonstrate that they meet requirements for adequate clinical space, secure data storage facilities, secure storage facilities for drugs and supplies, necessary computer hardware and software for data entry and transfer, spirometry

meeting American Thoracic Society standards, and adequate office space for staff and investigators.

Documentation: Clinics must file current copies of IRB approvals and conflict of interest statements with the DCC.

- Staff certification requirements

  Knowledge assessment: All study staff must complete a Knowledge Assessment form to demonstrate their understanding of the study protocol.

  Assurance statement: All study staff will sign a statement indicating their willingness to respect the privacy of participants and adhere to high standards of integrity in the collection, recording, and editing of data.

5. Protection of Human Subjects

5.1 Risks to the Participants

Participation in this research protocol requires routine clinical procedures that are more than minimal risk.

Bronchoscopy is a semi-invasive procedure that involves sedation, local anesthesia and insertion of a bronchoscope into the airways. Adverse events include epistaxis, laryngospasm, bronchospasm, hypoxemia, allergy or adverse reaction to the sedation, fever following the procedure, or infection. The most common adverse event is fever which occurs in approximately 5% of patients and is self-limited. Infection requiring antibiotics occurs in about 0.5% of patients. Some patients report mild chest soreness, hoarseness, or sore throat the day after the procedure, which is self-limited. During the FORTE study, 120 patients with COPD comparable to the severity included in this study undertook bronchoscopy and 1 patient required hospitalization following the procedure for bronchospasm. In the proposed study, we are performing BAL with 300 ml of saline which is more than some clinical procedures that use 100-150 ml of saline. Unless a patient has a history of bleeding tendency or we are planning to do a tissue biopsy, we do not routinely perform coagulation testing for bronchoscopy with lavage.

Pulmonary Function Testing requires deep and forceful respiratory efforts. It is a commonly performed and safe examination that is widely performed in patients with lung disorders. Some patients report chest soreness the day following the procedure. Some patients may experience light-headedness during the forced expiration. The risk of syncope is mitigated by having the patient perform the test in the seated rather than standing position.

During the testing, patients may be administered 4 inhalations of albuterol as a bronchodilator. This is higher than the FDA recommended dose of 2 inhalations, but is the recommended dose by the ATS/ERS for bronchodilator testing. Albuterol may cause a rapid heart rate, tremor, or palpitations. This risk is mitigated by checking the pulse rate prior to testing and requiring study physician approval to administer albuterol if the heart rate exceeds 110 bpm. If the patient has taken a short-acting bronchodilator within the past 4 hours then only 2 inhalations of albuterol are given.

During the diffusing capacity test, the patient is asked to breath in small (0.3%) concentrations of carbon monoxide which may be toxic or fatal if inhaled in larger concentrations or for prolonged periods of time. The risk of carbon monoxide toxicity is prevented by using medically certified gas concentrations, by analysis of the inhaled gas concentration prior to each test, and by limiting the number of tests to no more than 4 to obtain two replicates of good quality.

Nasal brushing is a safe procedure although it might induce a nosebleed or mild discomfort in susceptible individuals.

Blood draw / venipuncture can cause pain or discomfort and may leave a bruise.

Psychological or financial risks. Although unlikely, there may be unforeseen psychological or financial risks for participants in this trial. For example, participants may have unrealistic expectations of benefit from treatment received in a trial, or may have psychological distress from having a diagnosis of COPD or even other concomitant diseases discovered during screening evaluation. Participants may also have to undergo financial loss from their occupations to attend clinic visits. Most participants, however, receive psychological benefit from participating in a study that may help others, and the financial costs are mitigated by small honoraria for participation in the study.

5.2 Steps taken to minimize the risks.

Risks of bronchoscopy are minimized by having the patient fast for 8 hours prior to the procedure to avoid aspiration, performance of the procedure in a medically secure environment with trained personnel, continuous monitoring of oxygen saturation and vital signs during the procedure, monitoring the patient until full recovery in a secure environment after the procedure, and requiring a companion to accompany the participant home after the procedure with instructions not to drive until the next day.

Risks of pulmonary function testing are minimized by having the test done in the seated position and direct observation of the patient during the testing.

Risks of nasal brushing are minimized by doing the procedure with local lidocaine topical anesthesia and avoiding it in people who have had a recent nosebleed.

Risks of blood draw are minimized by limiting the amount drawn to no more than 60 ml at each visit where blood is drawn for a total maximum of 240 ml over 6 weeks. Patients have venipuncture in the seated or lying position to minimize syncopal events and pressure is applied after the procedure.

Legal risks such as the risks that would be associated with breach of confidentiality.
No highly confidential information is routinely collected, although the data collection does include private health information (PHI), so a breach of confidentiality would constitute a HIPAA violation. If a patient is harmed from this study as a consequence of negligence, then the investigators or University might be liable for damages.

Financial risks to the participants:
The participant is not charged for drugs or any of the study procedures. They may suffer financial loss from loss of work time to participate in the study. If a participant suffers an injury as a consequence of participation in this study, they would be responsible for the medical costs as the University and NIH do not have a program to reimburse patients for such costs.

Plan for reporting unanticipated problems or study deviations:
Unanticipated problems and protocol deviations are reported to the IRB, FDA, and NIH in accordance with their reporting requirements.

5.3 Recruitment and Consent Procedures

Participants will be recruited by each participating clinic by their own methods. These methods may include local campaigns, solicitation in physician offices, clinics, workplaces, schools and

public media advertisements. All public advertisements are subject to approval by the local IRB and must indicate that it is a research study. The DCC will help coordinate recruitment among clinics and promote sharing of effective recruitment strategies within the network. The trial will be registered on www.ClinicalTrials.gov.

Potential enrollees will be approached either in person, by telephone or by mail to establish general eligibility criteria. A general description of the study, including frequently asked questions and a consent form will be provided prior to their initial screening visit. If the potential enrollees are interested in attending a screening visit, they will meet with the study coordinator and the local physician co-investigators to review the study and answer questions. They will be asked to sign consent, and if appropriate provide assent, and undergo the screening procedures at that visit.

The consent form will be subject to approval by the clinical center IRB. A copy of the consent form will be given to each participant, and the signed original will be kept in the participant's research chart.

References

1. National Heart, Lung, and Blood Institute. Morbidity and mortality: 2009 chart book on cardiovascular, lung, and blood diseases. Available at: http://www.nhlbi.nih.gov/resources/docs/2009_ChartBook.pdf. Accessed January 18, 2010.

2. Puhan MA, Scharplatz M, Troosters T, Steurer J. Respiratory rehabilitation after acute exacerbation of COPD may reduce risk for readmission and mortality --a systematic review. Respir Res. 2005 Jun 8;6:54

3. Wedzicha, J.A., Seemungal TA, COPD exacerbations: defining their cause and prevention. Lancet, 2007. 370(9589): 786-96.

4. Mannino DM, Braman S. The epidemiology and economics of chronic obstructive pulmonary disease. Proc Am Thorac Soc. 2007 Oct 1;4(7):502-6

5. Langen RC, Korn SH, Wouters EF. ROS in the local and systemic pathogenesis of COPD. Free Radic Biol Med. 2003;35:226-35.

6. Bowler RP, Barnes PJ, Crapo JD. The role of oxidative stress in chronic obstructive pulmonary disease. COPD 2004;1:255–77.

7. MacNee W. Oxidants/antioxidants and COPD. Chest 2000; 117(suppl):303S–17S.

8. Taylor AE, Finney-Hayward TK, Quint JK, Thomas CM, Tudhope SJ, Wedzicha JA, Barnes PJ, Donnelly LE. Defective macrophage phagocytosis of bacteria in COPD. Eur Respir J. 2009 Nov 6. Epub

9. Berenson CS, Garlipp MA, Grove LJ, Maloney J, & Sethi S Impaired phagocytosis of nontypeable Haemophilus influenzae by human alveolar macrophages in chronic obstructive pulmonary disease. J Infect Dis 2006;194:1375-1384

10. Marwick JA, Ito K, Adcock IM, Kirkham PA. Oxidative stress and steroid resistance in asthma and COPD: pharmacological manipulation of HDAC-2 as a therapeutic strategy. Expert Opin Ther Targets. 2007;11:745-55

11. Van Eeden SF, Sin DD. Chronic obstructive pulmonary disease: a chronic systemic inflammatory disease. Respiration. 2008;75(2):224-38

12. Kim V, Rogers TJ, Criner GJ. New concepts in the pathobiology of chronic obstructive pulmonary disease. Proc Am Thorac Soc. 2008;5:478-85.

13. Man SF, Connett JE, Anthonisen NR, Wise RA, Tashkin DP, Sin DD. C-reactive protein and mortality in mild to moderate chronic obstructive pulmonary disease. Thorax. 2006;61:849-53.

14. Veeramachaneni, S.B. and S. Sethi, Pathogenesis of bacterial exacerbations of COPD. COPD, 2006. 3: p. 109-15.

15. Barnes PJ, Ito K, Adcock IM. Corticosteroid resistance in chronic obstructive pulmonary disease: inactivation of histone deacetylase. Lancet. 2004;363(9410):731-3.

16. Rahman I, van Schadewijk AA, Crowther AJ, Hiemstra PS, Stolk J, MacNee W, De Boer WI. 4-Hydroxy-2-nonenal, a specific lipid peroxidation product, is elevated in lungs of patients with chronic obstructive pulmonary disease. *Am J Respir Crit Care Med* 2002;166:490–495

17. Wright DT, Fischer BM, Li C, Rochelle LG, Akley NJ, Adler KB. Oxidant stress stimulates mucin secretion and PLC in airway epithelium via a nitric oxide-dependent mechanism. Am J Physiol. 1996; 271:L854-61.

18. McMaster SK, Paul-Clark MJ, Walters M, Fleet M, Anandarajah J, Sriskandan S, Mitchell JA. Cigarette smoke inhibits macrophage sensing of Gram-negative bacteria and lipopolysaccharide: relative roles of nicotine and oxidant stress. Br J Pharmacol. 2008;153:536-43.

19. Gong P, Stewart D, Hu B, Li N, Cook J, Nel A, Alam J. Activation of the mouse heme oxygenase-1 gene by 15-deoxy-Delta(12,14)-prostaglandin J(2) is mediated by the stress response elements and transcription factor Nrf2. *Antioxid Redox Signal* 2002;4:249–257

20. Bea F, Hudson FN, Chait A, Kavanagh TJ, Rosenfeld ME. Induction of glutathione synthesis in macrophages by oxidized low-density lipoproteins is mediated by consensus antioxidant response elements. Circ Res 2003;92:386–393.

21. Venugopal R, Jaiswal AK. Nrf1 and Nrf2 positively and c-Fos and Fra1 negatively regulate the human antioxidant response element-mediated expression of NAD(P)H:quinone oxidoreductase1 gene. Proc Natl Acad Sci USA 1996;93:14960–14965

22. Malhotra D, Thimmulappa R, Navas-Acien A, Sandford A, Elliott M, Singh A, Chen L, Zhuang X, Hogg J, Pare P, Tuder RM, Biswal S. Decline in NRF2-regulated antioxidants in chronic obstructive pulmonary disease lungs due to loss of its positive regulator, DJ-1. Am J Respir Crit Care Med. 2008;178:592-604.

23. Rangasamy T, Cho CY, Thimmulappa RK, Zhen L, Srisuma SS, Kensler TW, Yamamoto M, Petrache I, Tuder RM, Biswal S. Genetic ablation of Nrf2 enhances susceptibility to cigarette smoke-induced emphysema in mice. J Clin Invest 2004;114:1248–1259.

24. Blake DJ, Singh A, Kombairaju P, Malhotra D, Mariani TJ, Tuder RM, Gabrielson E, Biswal S. Deletion of Keap1 in the Lung Attenuates Acute Cigarette Smoke-induced Oxidative Stress and Inflammation. Am J Respir Cell Mol Biol. 2009 Jun 11. [Epub ahead of print]

25. Singh A, Rangasamy T, Thimmulappa RK, Lee H, Osburn WO, Brigelius-Flohe R, Kensler TW, Yamamoto M, Biswal S. Glutathione peroxidase 2, the major cigarette smoke-inducible isoform of GPX in lungs, is regulated by Nrf2. *Am J Respir Cell Mol Biol* 2006;35:639–650

26. Malhotra D, Thimmulappa R, Vij N, Navas-Acien A, Sussan T, Merali S, Zhang L, Kelsen SG, Myers A, Wise R, Tuder R, Biswal S. Heightened endoplasmic reticulum stress in the lungs of patients with chronic obstructive pulmonary disease: the role of Nrf2-regulated proteasomal activity. Am J Respir Crit Care Med. 2009;180:1196-207.

27. Decramer M, Rutten-van Molken M, Dekhuijzen PN, Troosters T, van Herwaarden C, Pellegrino R, van Schayck CP, Olivieri D, Del Donno M, De Backer W, et al. Effects of N-acetylcysteine on outcomes in chronic obstructive pulmonary disease (Bronchitis Randomized on NAC Cost-Utility Study, BRONCUS): a randomised placebo-controlled trial. Lancet 2005;365:1552–1560.

28. Mudway IS, Behndig AF, Helleday R, Pourazar J, Frew AJ, Kelly FJ, Blomberg A. Vitamin supplementation does not protect against symptoms in ozone-responsive subjects. Free Radic Biol Med 2006;40:1702–1712.

29. Oudshoorn JH, Klijn PH, Hofman Z, Voorbij HA, van der Ent CK, Berger R, Houwen RH. Dietary supplementation with multiple micronutrients: no beneficial effects in pediatric cystic fibrosis patients. J Cyst Fibros 2007;6:35–40.

30. Rahman I. Antioxidant therapeutic advances in COPD. Ther Adv Respir Dis. 2008;2:351-74.

31. Kwak, M.K., Itoh, K., Yamamoto, M., Sutter, T.R., and Kensler, T.W. 2001. Role of transcription factor Nrf2 in the induction of hepatic phase 2 and antioxidative enzymes in vivo by the cancer chemoprotective agent, 3H-1, 2-dimethiole-3-thione. *Mol Med* 7:135-145.

32. Dinkova, Kostova, A.T., Holtzclaw, W.D., Cole, R.N., Itoh, K., Wakabayashi, N., Katoh, Y., Yamamoto, M., and Talalay, P. 2002. Direct evidence that sulfhydryl groups of Keap1 are the sensors regulating induction of phase 2 enzymes that protect against carcinogens and oxidants. Proc Natl Acad Sci U S A 99:11908-11913.

33. Dinkova, Kostova, A.T., Liby, K.T., Stephenson, K.K., Holtzclaw, W.D., Gao, X., Suh, N., Williams, C., Risingsong, R., Honda, T., et al. 2005. Extremely potent triterpenoid inducers of the phase 2 response: correlations of protection against oxidant and inflammatory stress. Proc Natl Acad Sci U S A 102:4584-4589.

34. Kode, A., Rajendrasozhan, S., Caito, S., Yang, S.R., Megson, I.L., and Rahman, I. 2008. Resveratrol induces glutathione synthesis by activation of Nrf2 and protects against cigarette smoke-mediated oxidative stress in human lung epithelial cells. Am J Physiol Lung Cell Mol Physiol 294:L478-488.

35. Shen, G., Xu, C., Hu, R., Jain, M.R., Gopalkrishnan, A., Nair, S., Huang, M.T., Chan, J.Y., and Kong, A.N. 2006. Modulation of nuclear factor E2-related factor 2-mediated gene expression in mice liver and small intestine by cancer chemopreventive agent curcumin. Mol Cancer Ther 5:39-51.

36. Yao, P., Nussler, A., Liu, L., Hao, L., Song, F., Schirmeier, A., and Nussler, N. 2007. Quercetin protects human hepatocytes from ethanol-derived oxidative stress by inducing heme oxygenase-1 via the MAPK/Nrf2 pathways. J Hepatol 47:253-261.

37. Thimmulappa RK, Mai KH, Srisuma S, Kensler TW, Yamamoto M, Biswal S. Identification of Nrf2-regulated genes induced by the chemopreventive agent sulforaphane by oligonucleotide microarray. Cancer Res. 2002;62:5196-203.

38. Malhotra D, Thimmulappa R, Vij N, Navas-Acien A, Sussan T, Merali S, Zhang L, Kelsen SG, Myers A, Wise R, Tuder R, Biswal S. Heightened endoplasmic reticulum

stress in the lungs of patients with chronic obstructive pulmonary disease: the role of Nrf2-regulated proteasomal activity. Am J Respir Crit Care Med. 2009 Dec 15;180(12):1196-207.

39. Innamorato, N.G., Rojo, A.I., Garcia, Yague, A.J., Yamamoto, M., de Ceballos, M.L., and Cuadrado, A. 2008. The transcription factor Nrf2 is a therapeutic target against brain inflammation. J Immunol 181:680-689.

40. Cho, H.Y., Imani, F., Miller, DeGraff, L., Walters, D., Melendi, G.A., Yamamoto, M., Polack, F.P., and Kleeberger, S.R. 2009. Antiviral activity of Nrf2 in a murine model of respiratory syncytial virus disease. Am J Respir Crit Care Med 179:138-150

41. Zakkar, M., Van der Heiden, K., Luong le, A., Chaudhury, H., Cuhlmann, S., Hamdulay, S.S., Krams, R., Edirisinghe, I., Rahman, I., Carlsen, H., et al. 2009. Activation of Nrf2 in endothelial cells protects arteries from exhibiting a proinflammatory state. Arterioscler Thromb Vasc Biol 29:1851-1857.

42. Kong, J.S., Yoo, S.A., Kim, H.S., Kim, H.A., Yea, K., Ryu, S.H., Chung, Y.J., Cho, C.S., and Kim, W.U. Inhibition of synovial hyperplasia, rheumatoid T cell activation, and experimental arthritis in mice by sulforaphane, a naturally occurring isothiocyanate. Arthritis Rheum 62:159-170.

43. Piao CS, Gao S, Lee GH, Kim DS, Park BH, Chae SW, Chae HJ, Kim SH. Sulforaphane protects ischemic injury of hearts through antioxidant pathway and mitochondrial K(ATP) channels. Pharmacol Res. 2009 Dec 3. [Epub ahead of print]

44. Wu, L., Noyan Ashraf, M.H., Facci, M., Wang, R., Paterson, P.G., Ferrie, A., and Juurlink, B.H. 2004. Dietary approach to attenuate oxidative stress, hypertension, and inflammation in the cardiovascular system. Proc Natl Acad Sci U S A 101:7094-7099.

45. Zhao, X., Song, S., Sun, G., Strong, R., Zhang, J., Grotta, J.C., and Aronowski, J. 2009. Neuroprotective role of haptoglobin after intracerebral hemorrhage. J Neurosci 29:15819-15827.

46. Dash, P.K., Zhao, J., Orsi, S.A., Zhang, M., and Moore, A.N. 2009. Sulforaphane improves cognitive function administered following traumatic brain injury. Neurosci Lett 460:103-107.

47. Talalay, P., Fahey, J.W., Healy, Z.R., Wehage, S.L., Benedict, A.L., Min, C., Dinkova, and Kostova, A.T. 2007. Sulforaphane mobilizes cellular defenses that protect skin against damage by UV radiation. Proc Natl Acad Sci U S A 104:17500-17505.

48. Kerns, M.L., DePianto, D., Dinkova, Kostova, A.T., Talalay, P., and Coulombe, P.A. 2007. Reprogramming of keratin biosynthesis by sulforaphane restores skin integrity in epidermolysis bullosa simplex. Proc Natl Acad Sci U S A 104:14460-14465.

49. Fahey, J.W., Haristoy, X., Dolan, P.M., Kensler, T.W., Scholtus, I., Stephenson, K.K., Talalay, P., and Lozniewski, A. 2002. Sulforaphane inhibits extracellular, intracellular, and antibiotic-resistant strains of Helicobacter pylori and prevents benzo[a]pyrene-induced stomach tumors. Proc Natl Acad Sci U S A 99:7610-7615.

50. Conaway, C.C., Wang, C.X., Pittman, B., Yang, Y.M., Schwartz, J.E., Tian, D., McIntee, E.J., Hecht, S.S., and Chung, F.L. 2005. Phenethyl isothiocyanate and sulforaphane and their N-acetylcysteine conjugates inhibit malignant progression of lung adenomas induced by tobacco carcinogens in A/J mice. Cancer Res 65:8548-8557.

51. Zhang, Y., Kensler, T.W., Cho, C.G., Posner, G.H., and Talalay, P. 1994. Anticarcinogenic activities of sulforaphane and structurally related synthetic norbornyl isothiocyanates. Proc Natl Acad Sci U S A 91:3147-3150.

52. Salvi SS, Barnes PJ. Chronic obstructive pulmonary disease in non-smokers. Lancet. 2009 Aug 29;374(9691):733-43.

53. Mahler DA, Wells CK. Evaluation of clinical methods for rating dyspnea. Chest. 1988;93:580-6.

54. Meguro M, Barley EA, Spencer S, Jones PW. Development and Validation of an Improved, COPD-Specific Version of the St. George Respiratory Questionnaire. Chest. 2007;132:456-63

55. Shapiro TA, J.W. Fahey, K.L. Wade, K.K. Stephenson, P. Talalay, Chemoprotective glucosinolates and isothiocyanates of broccoli sprouts: metabolism and excretion in humans, Cancer Epidemiol. Biomark. Prev. 2001; 10: 501–508

56. Zhang Y, K.L. Wade, T. Prestera, P. Talalay, Quantitative determination of isothiocyanates, dithiocarbamates, carbon disulfide, and related thiocarbonyl compounds by cyclocondensation with 1,2-benzenedithiol, Anal. Biochem. 1996; 239:160–167.
