# Supplementary material for: Lack of Effect of Oral Sulforaphane Administration on Nrf2 Expression in COPD: A Randomized, Double-Blind, Placebo Controlled Trial
Source: PLoS One. 2016 Nov 10;11(11):e0163716. doi: 10.1371/journal.pone.0163716 (PMC5104323; doi:10.1371/journal.pone.0163716)
Supplement: S1 File — (PDF) [file pone.0163716.s003.pdf]

S1 File: Specimen Processing Methods

Bronchoalveolar Lavage Processing and cell separation

BAL fluid sample was filtered through sterile gauze into a sterile 50ml falcon tube to remove mucus plugs and centrifuged at 500g at 4 degrees for 10 minutes. The supernatant was decanted and cell pellet was re-suspended in 10 mL of ice-cold Hanks Buffered Saline Solution (HBSS) and centrifuged at 500g at 4 degrees for 10 minutes (repeated twice). After washing, cell pellet was re-suspended in 10ml of RPMI media supplemented with 10% FBS and cell count was performed using Adam MC automatic cell counter. Later the cells suspended in RPMI media was transferred into sterile 10 cm culture dish and incubate at 37^0C with 5% CO2 for 1 hour to let macrophages adhere. After 1hr, media was aspirated into a 15 mL conical tube to collect non-adherent cells. Wash the adherent cells twice with warm HBSS (gently add the media, swirl the culture dish for 4-5 times and remove the media) and 5 mL of Cell detachment buffer was added to the dish and incubated for 3 min at 37^0C. Adherent cells were gently scraped using a rubber cell scraper, transferred to a sterile 15 mL Falcon tube and the sample was centrifuge at 500xg at 4 degrees for 10 min. After decanting the supernatant, cell pellet was re-suspended in 3 mL of RPMI media without FBS and centrifuged 500xg at 4 degrees for 10 min. Supernatant was discarded and RLT lysis buffer was added to the pellet for gene expression analysis

Serum separation

Blood was collected in two yellow top, blood collection tubes (BD SST™ Hemogard vacutainers, cat # 367986). The tubes should sit upright after the blood is drawn at room temperature for a minimum of 30 to a maximum of 60 minutes to allow the clot to form. The blood sample was centrifuged at the end of the clotting time (30-60 minutes) in a horizontal rotor (swing-out head) for 20 minutes at 1100-1300g at room temperature. Immediately after centrifugation, serum was transferred into 1.5 mL tubes and stored at -80C.

Plasma separation

Blood was collected into two sodium heparin coated 6 ml tubes (BD Hemogard™, BD Vacutainer, green top, catalog # 367878). After collection, the blood was gently mixed by inverting the tube 8 to 10 times and stored upright at 4^oC until centrifugation (Blood samples were centrifuged within 1 hour of blood collection). Blood sample was centrifuged in a horizontal rotor (swing-out head) for 20 minutes at 1200 g at room temperature. After centrifugation,

plasma layer which will be at the top of the tube was transferred into 1.5 mL tubes and stored at -80°C.

Peripheral blood mononuclear cell (PBMC) separation

Blood was collected via venipuncture directly into two CPT tubes (each CPT tube will hold ~8 mL blood) (BD Vacutainer® CPT™ Tube, Cat # 362753). After collection, tube was stored upright at room temperature until centrifugation. Blood samples were centrifuged within 1 hour of blood collection for best results. Blood sample was mixed immediately prior to centrifugation by gently inverting the tube 8 to 10 times and CPT tubes are centrifuged at 1800 g (approximately 2800 rpm on a Sorvall RT6000 centrifuge) for 30 minutes at room temperature. After centrifugation, mononuclear cells and platelets which will be in a whitish layer just under the plasma layer was collected with a Pasteur pipette immediately and transferred to a 50 mL falcon tubes. The cells were suspended in PBS, mixed and centrifuged for 8 minutes at 500g and supernatant was decanted without disturbing cell pellet. If there are appreciable amount of RBCs in the PBMC pellet, RBC lysis step was carried out. The cell pellet was re-suspended, mixed in 10 ml HBSS and cell count was performed using Adam MC automatic cell counter. The sample was centrifuged and cell pellet was re-suspended in 6 mL of RPMI 1640 medium. The cell suspension was aliquotted into different tubes and centrifuged at 500g. Supernatant was decanted and RLT lysis buffer was added to one of the pellets for gene expression analysis. The other aliquots were used for enzyme activity and functional analysis.

Nasal brushings

Nasal brush was introduced into 15 ml falcon tube containing 10 mL PBS and vigorously shaken to release the cells. After passing through cell strainer, the solution was centrifuged at 2050xg. Supernatant was discarded and RLT lysis buffer was added to the pellet for gene expression analysis.

Bronchial brushings

Tube was vortexed gently and rinsed vigorously into PBS to release the cells from the brush. After passing through cell strainer, the solution was centrifuged at 2050xg. Supernatant was discarded and RLT lysis buffer was added to the pellet for gene expression analysis.

Measurement of sulforaphane bioavailability

Sulforaphane absorption was predicted from the levels of its chief dithiocarbamate metabolites in the plasma as described previously[23]. Briefly, 500 µL of previously frozen plasma was

centrifuged (12,000 rpm on a tabletop micro-centrifuge, for 10 minutes at 4 °C), and 320 µL of supernatant was recovered and utilized in the cyclocondensation assay. Recoveries ranged from 0 to 474 pmol cyclocondensation product per mL of clarified plasma.

ELISA

Levels of IL-6 and IL-8 in serum and BAL fluid were measured by enzyme immunoassays using human ELISA kits (R&D systems) and quantified as per the manufacturer instructions. C-reactive protein (CRP) levels in serum were measured through a commercially available high sensitivity kit (Helica Biosystems, Santa Ana, CA).

Oxidative stress markers

Isoprostane: 8-isoprostane levels were measured in exhaled breath condensate samples with a commercially available enzyme immunoassay kit (Cayman Chemical, Ann Arbor, MI, USA) with a lower detection limit of 2.7 pg/mL. Briefly, the samples were purified through SPE columns and assay was performed as per the manufacturer's instructions. Plasma isoprostane was measured using commercially available kit (Cell Biolabs, San Diego, CA).

BAL differential count: The BAL fluid was immediately centrifuged at 1,500 *g*. The total cell count was measured, and cytospin preparation (Shandon Scientific Inc.) was performed. Cells were stained with Diff-Quick reagent (Baxter Dade), and a differential count of 200 cells was performed using standard morphological criteria.

TBARS: Thiobarbituric acid reactive substances (TBARS) were measured in serum using commercially available kit (Cell Biolabs). Briefly, Serum samples were mixed with SDS lysis buffer at a ratio of 1:1 followed by addition of thiobarbituric acid. Samples were boiled, then mixed with n-butanol at a ratio of 1:1, and absorbance was measured at 532 nm. Malondialdehyde content was compared to a standard curve.

Total antioxidant capacity: The total antioxidant capacity in plasma was measured using Cayman's antioxidant assay kit (Cayman Chemical Company, Ann Arbor, MI, USA). The assay relies on the ability of the antioxidants in the sample to inhibit the oxidation of ABTS [2,2′-Azino-di-(3-ethylbenthiazoline sulphonate)] to ABTS+ by metmyoglobin that was detected spectrophotometrically at 405 nm. The capacity of antioxidants in the sample to prevent the oxidation of ABTS was compared with that of Trolox, a water-soluble tocopherol analog and the results are expressed as millimolar Trolox equivalents.
