# Supplementary material for: Lack of Effect of Oral Sulforaphane Administration on Nrf2 Expression in COPD: A Randomized, Double-Blind, Placebo Controlled Trial
Source: PLoS One. 2016 Nov 10;11(11):e0163716. doi: 10.1371/journal.pone.0163716 (PMC5104323; doi:10.1371/journal.pone.0163716)
Supplement: S1 Table — (PDF) [file pone.0163716.s004.pdf]

S1 Table: Hematology and serum chemistry at baseline and change after 4 weeks by treatment assignment

	Sulforaphane Dose Group			
	Placebo N=30	***25μM N=29***	***150μ N=29***	***P-value****
	Median (Interquartile Range)			
Hemoglobin (g/dL)				
Baseline	14.9 (13.0,15.4)	14.9 (14.1,15.6)	14.3 (13.5,14.9)	
Change from baseline	0.0 (-0.4,0.6)	0.0 (-0.5,0.2)	-0.1 (-0.4,0.4)	0.68
Hematocrit (%)				
Baseline	44 (39,44)	44 (42,46)	42 (41,44)	
Change from baseline	0 (-1,2)	-0 (-2,1)	-0 (-1,2)	0.41
White blood cell count (x10^4/mL)				
Baseline	0.7 (0.5,0.9)	0.7 (0.6,0.8)	0.6 (0.5,0.7)	
Change from baseline	0.0 (-0.2,0.0)	0.0 (-0.1,0.0)	0.0 (-0.1,0.1)	0.45
Thyroid stimulating hormone (μ U/mL)				
Baseline	1.1 (0.7,1.6)	1.2 (0.5,1.6)	1.1 (0.8,1.7)	
Change from baseline	0.0 (-0.4,0.3)	0.0 (-0.2,0.1)	0.1 (-0.3,0.6)	0.46
Aspartate transaminase (AST) (U/L)				
Baseline	19 (17,23)	20 (15,29)	21 (18,29)	
Change from baseline	0 (-2,5)	-2 (-3,2)	0 (-3,2)	0.48
Alanine transaminase (ALT) (U/L)				
Baseline	19 (15,26)	24 (18,31)	21 (18,32)	
Change from baseline	0 (-1,3)	0 (-5,1)	1 (-4,4)	0.28
Alkaline phosphatase (U/L)				
Baseline	84 (72,90)	75 (68,93)	77 (66,87)	
Change from baseline	2 (-6,7)	-2 (-5,4)	-2 (-6,8)	0.82
Creatinine (mg/dL)				
Baseline	0.80 (0.80,1.00)	1.00 (0.70,1.00)	0.90 (0.80,1.10)	
Change from baseline	0.00 (0.00,0.10)	0.00 (-0.10,0.10)	0.10 (-0.10,0.10)	0.47

*Kruskal-Wallis test
