# Supplementary material for: Lack of Effect of Oral Sulforaphane Administration on Nrf2 Expression in COPD: A Randomized, Double-Blind, Placebo Controlled Trial
Source: PLoS One. 2016 Nov 10;11(11):e0163716. doi: 10.1371/journal.pone.0163716 (PMC5104323; doi:10.1371/journal.pone.0163716)
Supplement: S2 Table — (PDF) [file pone.0163716.s005.pdf]

S2 Table: Pulmonary function measures, change from baseline to 4 weeks by treatment assignment

	Sulforaphane Dose Group			
	Placebo N=30	***25μM N=29***	***150μM N=29***	***P-value****
	Median (Interquartile Range)			
Post bronchodilator FEV1 (%predicted)	-1 (-4, 5)	1 (-4, 4)	1 (-4, 3)	0.83
Post bronchodilator FEV1/FVC ratio	-0.00 (-0.02, 0.02)	0.01 (-0.02, 0.02)	-0.00 (-0.03, 0.01)	0.71
DLCO (mL/mm/mmHg)	-0.2 (-1.8, 1.6)	-0.2 (-1.6, 1.9)	0.0 (-1.3, 1.1)	0.96
TLC (Liters)	-0.0 (-0.4, 0.2)	0.0 (-0.4, 0.2)	0.0 (-0.3, 0.3)	0.96
SVC (Liters)	0.1 (-0.3, 0.3)	0.0 (-0.3, 0.2)	0.0 (-0.3, 0.2)	0.78
FRC (Liters)	0.0 (-0.3, 0.2)	-0.0 (-0.2, 0.3)	0.0 (-0.1, 0.3)	0.66
RV (Liters)	-0.1 (-0.3, 0.2)	-0.1 (-0.4, 0.3)	0.1 (-0.4, 0.3)	0.81

Abbreviations: FEV1=forced expiratory volume in 1 second; FVC=forced vital capacity; DLCO=diffusing capacity; TLC=total lung capacity; SVC=slow vital capacity; FRC=forced residual capacity; RV =residual volume
*Kruskal-Wallis test
