# Supplementary material for: Lack of Effect of Oral Sulforaphane Administration on Nrf2 Expression in COPD: A Randomized, Double-Blind, Placebo Controlled Trial
Source: PLoS One. 2016 Nov 10;11(11):e0163716. doi: 10.1371/journal.pone.0163716 (PMC5104323; doi:10.1371/journal.pone.0163716)
Supplement: S3 Table — (PDF) [file pone.0163716.s006.pdf]

S3 Table: Dyspnea and St. George Respiratory Questionnaire Scores, change from baseline at 4 weeks by treatment assignment

	Sulforaphane Dose Group			
	Placebo N=30	***25μM N=29***	***150μM N=29***	***P-value****
	Median (Interquartile Range)			
Medical Research Council Dyspnea Score	0.0 (0.0,0.0)	0.0 (-1.0,0.0)	0.0 (0.0,1.0)	0.01
St. George's Respiratory Questionnaire				
Total score	-1.3 (-7.4,0.8)	1.0 (-4.1,3.8)	-3.5 (-6.6,1.8)	0.37
Symptoms	-1.1 (-9.4,4.5)	-1.6 (-12.1,6.2)	0.0 (-11.2,7.9)	0.88
Activity	0.0 (-12.7,6.7)	0.0 (-6.1,6.7)	0.0 (-6.3,6.6)	0.88
Impacts	-2.5 (-7.3,1.5)	1.9 (-3.9,7.2)	-1.9 (-6.9,3.5)	0.25

*Kruskal-Wallis test
