# Supplementary material for: Lack of Effect of Oral Sulforaphane Administration on Nrf2 Expression in COPD: A Randomized, Double-Blind, Placebo Controlled Trial
Source: PLoS One. 2016 Nov 10;11(11):e0163716. doi: 10.1371/journal.pone.0163716 (PMC5104323; doi:10.1371/journal.pone.0163716)
Supplement: S4 Table — (PDF) [file pone.0163716.s007.pdf]

S4 Table: Treatment-emergent† symptoms during follow-up by treatment group

Symptom	*Sulforaphane Dose Group* Placebo N=31	25µM N=29	150µM N=29	*P-value**
		N (%)		
Nausea	1 (3%)	2 (7%)	6 (21%)	0.09
Vomiting	0 (0%)	1 (3%)	0 (0%)	0.66
Poor appetite	1 (3%)	2 (7%)	2 (7%)	0.74
Bad taste in mouth	2 (7%)	7 (24%)	9 (31%)	0.05
Heartburn	1 (3%)	6 (21%)	7 (24%)	0.06
Headache	2 (7%)	2 (7%)	1 (3%)	1.00
Fatigue	3 (10%)	5 (17%)	2 (7%)	0.50
Skin rash	1 (3%)	2 (7%)	1 (3%)	0.84
Bloating/gas	5 (17%)	7 (24%)	6 (21%)	0.76
Diarrhea	2 (7%)	3 (10%)	2 (7%)	0.89
Abdominal discomfort	1 (3%)	3 (10%)	6 (21%)	0.09
Respiratory infection	1 (3%)	1 (3%)	1 (3%)	1.00
Chest pain	0 (0%)	2 (7%)	0 (0%)	0.21
Constipation	0 (0%)	1 (3%)	1 (3%)	0.55
Increased phlegm	1 (3%)	0 (0%)	1 (3%)	1.00
Leg pain	0 (0%)	1 (3%)	1 (3%)	0.55
Shortness of breath	0 (0%)	1 (3%)	1 (3%)	0.55
Other	4 (13%)	4 (14%)	0 (0%)	0.12

* P-value from Fisher's Exact test

†Emergent (new) symptoms reported as mild, moderate, or severe during the 4 weeks of follow-up.

Only bad taste (n=1) and heartburn (n=1) were characterized as severe.
